# Double Excitation Energies from Quantum Monte Carlo Using State-Specific Energy Optimization

**DOI:** 10.1021/acs.jctc.2c00769

**Published:** 2022-10-31

**Authors:** Stuart Shepard, Ramón L. Panadés-Barrueta, Saverio Moroni, Anthony Scemama, Claudia Filippi

**Affiliations:** † MESA+ Institute for Nanotechnology, University of Twente, 7500 AE Enschede, The Netherlands; ‡ CNR-IOM DEMOCRITOS, Istituto Officina dei Materiali and SISSA Scuola Internazionale Superiore di Studi Avanzati, Via Bonomea 265, I-34136 Trieste, Italy; § Laboratoire de Chimie et Physique Quantiques, Université de Toulouse, CNRS, UPS, 31062 Toulouse, France

## Abstract

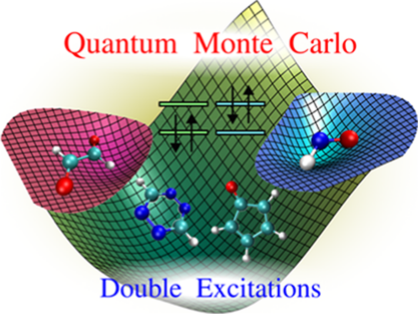

We show that recently developed quantum Monte Carlo methods, which provide accurate vertical transition energies for single excitations, also successfully treat double excitations. We study the double excitations in medium-sized molecules, some of which are challenging for high-level coupled-cluster calculations to model accurately. Our fixed-node diffusion Monte Carlo excitation energies are in very good agreement with reliable benchmarks, when available, and provide accurate predictions for excitation energies of difficult systems where reference values are lacking.

## 1 Introduction

An open challenge in computational chemistry is to develop a method which can accurately treat different types of excited states on an equal footing at an affordable cost. An effective way to assess the accuracy of different methods is by comparing vertical transition energies (VTEs) with the theoretical best estimates (TBEs) determined from high-level computational methods. Environmental effects can influence the measured transition energy in the experiment while VTEs calculated using the same molecular geometry are directly comparable.

Conventional single-reference methods, such as time-dependent density functional theory (TD-DFT)^1−3^ and coupled-cluster (CC) theories (equation-of-motion/linear response^4^), struggle to model double excitations, that is, excitations whose determinant expansions are dominated by determinants having two orbitals differing from a given reference determinant. The most commonly used, best-scaling, version of TD-DFT uses a linear response formalism which cannot treat multi-electron excitations. A relatively uniform description of singly and doubly excited states relies on workarounds and ad-hoc choices of the exchange–correlation functional.^5−10^

In theory, CC can capture double excitations, but in practice the excitation level must be truncated. For an *M*-electron excitation, CC must include at least *M* + 1 excitations to include a satisfactory amount of correlation,^11^ but often the *M* + 2 level theory is needed to obtain reasonable excitation energies.^12−16^ CC theories needed to treat single and double excitations such as CC3^17^ and CCSDT^18^ (for singles), and CC4^19^ and CCSDTQ^20^ (for doubles) scale poorly with system size as *N*^7^, *N*^8^, *N*^9^, and *N*^10^, respectively, (with *N* the number of electrons) limiting their application primarily to small molecules.

Multi-reference methods such as complete active space self-consistent field (CASSCF), CASSCF with a second-order perturbation energy correction (CASPT2),^21^ and the second-order *n*-electron valence state perturbation theory (NEVPT2)^22^ are better suited to treat double excitations. Unfortunately, these methods scale exponentially with the number of orbitals and electrons in the active space, limiting them to small active spaces and system size. CASPT2 tends to underestimate the VTEs of organic molecules, and a shift is generally introduced in the zeroth-order Hamiltonian to provide better global agreement.^23^ These multi-reference methods also rely on chemical intuition to choose which orbitals to include in the active space, but it is often unintuitive which active space will capture the important determinants of a given state.

Selected configuration interaction (sCI)^24,25^ methods are capable of obtaining double excitation energies and have recently been shown to reach full-CI (FCI) quality energies for small molecules.^26^ Among these approaches is the CI perturbatively selected iteratively (CIPSI)^24^ method in which determinants are selected based on their contribution to the second-order perturbation (PT2) energy, so that the most energetically relevant determinants are included in the determinant expansion first. This selection criterion both circumvents the excitation-ordered exponential expansion of the wave function used by CI and CC methods and removes the dependence on one’s chemical intuition to decide which determinants to include in the expansion.

Quantum Monte Carlo (QMC) methods, specifically, variational (VMC) and fixed-node diffusion Monte Carlo (DMC) are promising first-principles approaches for solving the Schrödinger equation stochastically. These methods scale favorably with system size as *O*(*N*^4^) and naturally parallelize.^27−29^ In addition, recent improvements in QMC algorithms^30−33^ allow for fast optimization of trial wave functions with thousands of parameters at a cost per Monte Carlo step of *O*(*N*^3^) + *O*(*N*_det_),^32^ where *N*_det_ is the number of determinants in the wave function. When using multi-determinant expansions, provided by the CIPSI method, fully optimized in the presence of a Jastrow factor as trial wave functions, VMC and DMC have been shown to provide accurate excitation energies.^34−36^ The role of the Jastrow factor is to account for dynamic correlations allowing for even shorter determinant expansions.^37−44^

It is shown here that the same VMC and DMC protocols, used previously to calculate single excitations,^36^ can be used to treat double excitations just as precisely. The pure double excitations of nitroxyl, glyoxal, and tetrazine are calculated using VMC and DMC. In addition, a prediction is made for two excitation energies in cyclopentadienone which have strong double-excitation character in both excited states. The trial wave functions of the ground and excited states are optimized simultaneously, maintaining orthogonality on-the-fly by imposing an overlap penalty.

## 2 Methods

In the following, we present how we build the trial wave functions for the QMC calculations and how we optimize them in VMC energy minimization using a penalty-based, state-specific scheme for excited states, similar to previous approaches.^45−47^

### 2.1 Wave Functions

The QMC trial wave functions take the Jastrow–Slater form
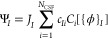
1where *C*_*i*_ are spin-adapted configuration state functions (CSFs), *c*_*Ii*_ are the expansion coefficients, *J*_*I*_ are the Jastrow factors, which include electron−electron and electron−nucleus pair correlations, and {ϕ}_*I*_ are the one-particle orbitals. In all calculations presented here, the ground and excited states have the same symmetry, so the CSFs, *C*_*i*_ , share the same orbital occupation patterns for all states. Note that each state, *I*, in eq 1, has its own optimal Jastrow factor, set of orbitals, and expansion coefficients. When each state has its own optimizable Jastrow and orbital parameters, the approach is referred to as a state-specific optimization, as opposed to a state-average optimization where all states share a common Jastrow factor and set of orbitals, and only the expansion coefficients are state-dependent.^35,37^

The expansion coefficients and orbitals are initialized for the VMC optimization by one of the two methods: (i) a *N*_state_-state-average [SA(*N*_state_)] CASSCF calculation or (ii) a CIPSI calculation, which builds off of approach (i). In approach (i), the SA-CASSCF wave functions are used as starting determinant components in the VMC optimization. In approach (ii), one only utilizes the orbitals produced by the SA-CASSCF calculation as the molecular orbital basis for a CIPSI expansion; in the VMC optimization, the starting determinant components include the CASSCF orbitals and CIPSI coefficients, *c*_*Ii*_.

Alternatively, in approach (ii), the natural orbitals (NO) are calculated from a first CIPSI expansion followed by a second CIPSI calculation in the NO basis. Performing a CIPSI expansion in the NO basis tends to lead to a smoother convergence to lower energies with fewer determinants^14^ compared to using a basis of SA-CASSCF orbitals. In other words, one can obtain a higher quality wave function with fewer determinants *via* relaxation of the molecular orbital basis.

Further details on the CASSCF initial wave functions and CIPSI calculations using the NO basis are provided in the Supporting Information.

### 2.2 Penalty-Based State-Specific Method

When trying to optimize a state which is not the lowest in its symmetry class, the state can collapse to a lower-energy eigenstate. A number of approaches have already been proposed to remedy this issue within QMC, including state-average energy minimization^37^ and state-specific variance minimization.^35^ The former requires that all states share a common Jastrow factor and set of orbitals. While the state-average approach has previously been shown to provide accurate results for excited states,^36,37^ the shared Jastrow factor and orbitals naturally compromise the flexibility of the wave functions. The latter approach^48−51^ is attractive as it avoids the non-variationality of the excited state energy and the need for extra constraints but, during long optimizations, it was found that one may lose the state of interest due to shallow or no minima in the associated functional.^35^

Another option to avoid collapse of the excited state is to use state-specific energy minimization with constraints. One way to do this is to impose orthogonality between the higher energy state and all states lower in energy. To this end, we employ a penalty-based state-specific method which requires minimizing the objective function^47^
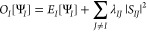
2where *E*_*I*_ is given by
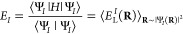
3*E*_L_^*I*^ is the local energy, *H*Ψ_*I*_/Ψ_*I*_, and ⟨·⟩_**R**∼·_ denotes the Monte Carlo average of the quantity in brackets over the electron configurations, **R**, sampled from the indicated distribution (in this case, |Ψ_I_(**R**)|^2^). The normalized overlap, *S*_*IJ*_, is given by
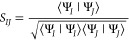
4

In order to simultaneously sample quantities for multiple states with comparable efficiency, a guiding function, , is introduced (see the Supporting Information for the calculation of γ_*I*_), and the energy of a given state (eq 3) is estimated as a weighted average
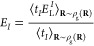
5where now the configurations are sampled from the distribution, ρ_g_ = |Ψ_g_|^2^, and the weight *t*_*I*_ is given by *t*_*I*_ = |Ψ_*I*_/Ψ_g_|^2^. Similar weighted averages are calculated for the derivatives of each state (see the Supporting Information).

The Jastrow, orbital, and CSF parameters of each state are optimized using the stochastic reconfiguration (SR)^52,53^ method. The gradients used in the SR equations are found by taking the parameter derivatives of eq 2, also derived in the Supporting Information.

In the implementation proposed by Pathak *et al.*,^47^ the state being optimized, Ψ_*I*_, is forced to be orthogonal to fixed, pre-optimized, lower-energy states, or anchor states, Ψ_*J*_. Each higher-energy state is then optimized consecutively, imposing orthogonality with all anchor states lower in energy. Each λ_*IJ*_ in eq 2 controls the overlap penalty for the state currently being optimized, Ψ_*I*_, due to each anchor state, Ψ_*J*_. The value of λ_*IJ*_ is suggested to be on the order of, and larger than, the energy spacing between the states.

The method implemented here optimizes all states at the same time with orthogonality imposed on-the-fly. Since all states are changing from one SR step to the next, the overlap penalty must be imposed between all states. Therefore, “≠” is used in the summation in eq 2 instead of “<” so that each state is kept orthogonal to all states, even to those higher in energy. Importantly, in circumstances where states are close in energy, their order may change throughout the optimization and, with “≠” in eq 2, the ordering of the states in energy does not need to be known in advance. Finally, the concomitant optimization of all states leads to a reduced computational cost compared to ref (47) and benefits from the use of correlated sampling in the optimization itself.

### 2.3 Analysis of λ_*IJ*_’s Effect on Energies and VTEs

We illustrate the effect of λ_*IJ*_ on the optimization of the three-state cyclopentadienone system with a small CIPSI expansion on CAS orbitals, performing a total of 800 SR iterations for different choices λ^(*i*)^, each corresponding to a different triplet of values (λ_12_, λ_13_, λ_23_). In general, we expect that if the λ_*IJ*_ are too small, some of the states may lose orthogonality and collapse onto each other; on the other hand, if they are too large, the fluctuations of the overlaps *S*_*IJ*_ will be amplified, downgrading the efficiency of the SR minimization because more sampling will be required to pin down the optimal variational parameters at the desired level of statistical precision.

**Figure 1:**
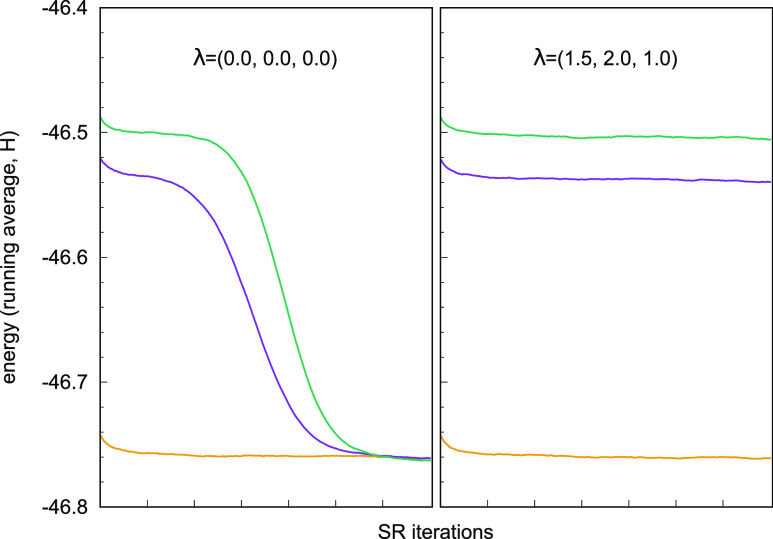
Plot of the VMC energy during optimization of the three-state cyclopentadienone system with a CIPSI wave function with 3036 determinants expanded on CAS orbitals. (Left) Collapse of the excited states to the ground state when λ_*IJ*_ = 0. (Right) Stable optimization using λ_12_ = 1.5, λ_13_ = 2.0, and λ_23_ = 1.0. The energy points in the plot are calculated from a running average of 100 SR iterations.

As shown in Figure 1, if all λ_*IJ*_ are set to zero, the excited states collapse toward the ground-state wave function (left panel), whereas they are bounded by the respective eigenstates with the choice of λ_*IJ*_ indicated in the right panel.

**Figure 2:**
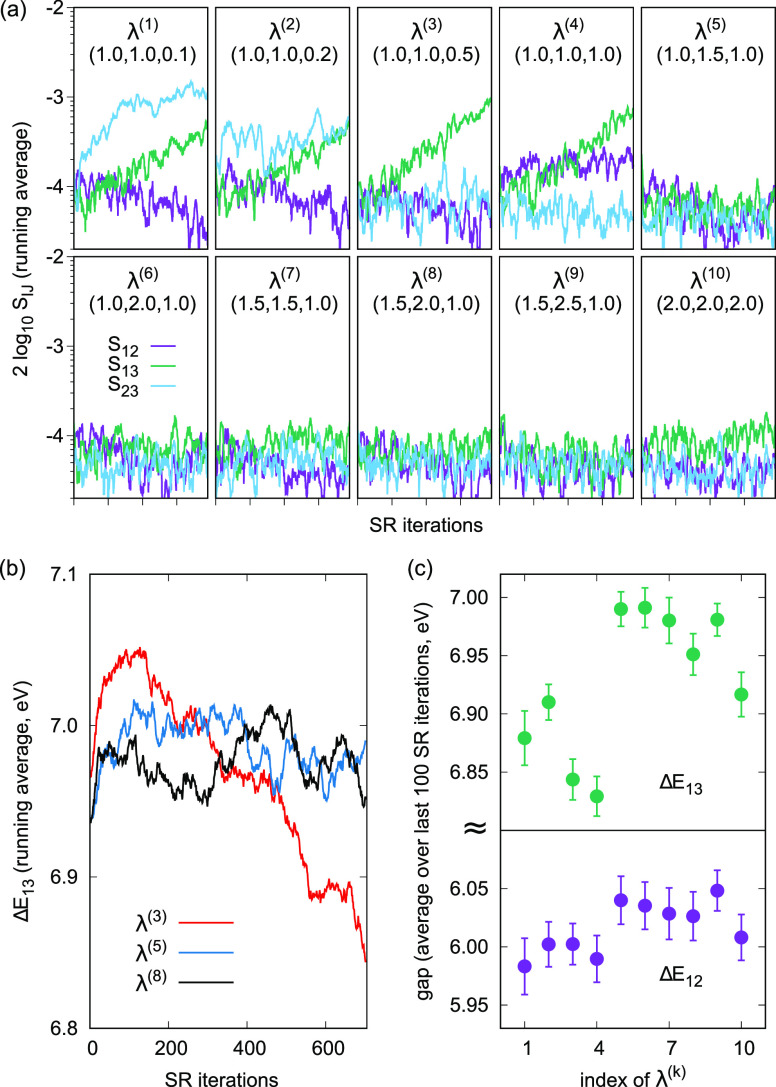
Effect of λ_*IJ*_ on the VMC optimization of the three-state cyclopentadienone system with a CIPSI wave function with 3036 determinants expressed on CAS orbitals. (a) Logarithmic plot of *S*_*IJ*_^2^ at each SR iteration for different λ^(*k*)^ = (λ_12_, λ_13_, λ_23_). (b) Running average (100 iterations) of Δ*E*_13_ for three different λ^(*k*)^. (c) Excitations energies averaged over the last 100 SR iterations for each λ^(*k*)^.

The selection of suitable values of λ_*IJ*_ is neither difficult nor critical. Figure 2 shows the overlaps |*S*_*IJ*_|^2^ and excitation energies (Δ*E*_*IJ*_) for various choices of λ_*IJ*_. In panel (a), for λ^(1)–(4)^, there is a noticeable increase in the overlaps, while the choices of λ^(5)–(9)^ prevent the overlaps from increasing; however, for λ^(10)^, |*S*_13_|^2^ tends to be uniformly larger. Panel (b) shows some examples of the correspondence between increasing overlap and decreasing excitation energy as well as cases where both are stable. Finally, panel (c) shows that all choices λ^(5)^–λ^(9)^ are equally good in terms of the final estimate of the excitation energies, whereas for λ^(1)^–λ^(4)^, they (particularly Δ*E*_13_) are somewhat smaller and may eventually collapse with more SR iterations. In the case of λ^(10)^, the signal is stable along the iterations, but the excitation energy Δ*E*_13_ is somewhat less accurate than that with λ^(5)^–λ^(9)^; it could be improved with more sampling per SR step, losing, however, efficiency.

In conclusion, there is a wide range of λ_*IJ*_ (λ^(5)^–λ^(9)^) where the estimate of the excitation energies is stable, consistent, and efficient.

In general, the nature of each individual excited state and the trial function itself will impact its sensitivity to the value of λ_*IJ*_. For instance, in the nitroxyl test case with the two-determinant wave function (see the Supporting Information), even with λ_*IJ*_ = 0, the excited state still cannot collapse completely to the ground state due to orbital symmetries. We also perform an analysis of a three-state optimization of nitroxyl (see the Supporting Information) which reveals little dependence of the VTEs on the choice of λ_*IJ*_.

As a final note, the number of unique values of λ_*IJ*_ grows as *N*_state_(*N*_state_ – 1)/2 and, without a formal way to determine appropriate values, the process above can in principle become time-consuming. However, based on the present work, we find that monitoring the overlaps and adjusting λ_*IJ*_ appropriately are effective means to ensure a stable optimization. This can of course also be done in an automated manner.

## 3 Computational Details

The geometries of nitroxyl, glyoxal, tetrazine, and cyclopentadienone are obtained from ground-state optimizations at the level of CC3/aug-cc-pVTZ.^12,13^ In all calculations, we utilize scalar-relativistic energy-consistent Hartree Fock (HF) pseudopotentials^54,64^ with the corresponding aug-cc-pVDZ Gaussian basis sets. The exponents of the diffuse functions are taken from the corresponding all-electron Dunning’s correlation-consistent basis sets.^55^ For glyoxal, we also tested the use of an aug-cc-pVTZ basis set and found compatible excitation energies both at the VMC and DMC level. All HF and SA-CASSCF calculations are performed in GAMESS(US)^56^ with equal weights on all states.

CIPSI calculations are performed with the Quantum Package^57,58^ program. All states of interest are singlets, so determinants are chosen such that the expansions remain eigenstates of *Ŝ*^2^ with eigenvalue 0. For each molecule, the ground and excited states of interest have the same symmetry so the same set of orbitals are used in all state’s determinant expansions. States are weighted, and determinants are added to the CIPSI expansion such that the PT2 energy and variance of all states remain similar as this has been shown to give more accurate QMC excitation energies.^34−36^ The choice of weights in the CIPSI selection criterion is detailed for each molecule in the Supporting Information.

QMC calculations are performed with the CHAMP^59^ program using the method detailed in Section 2.2. Damping parameters ranging from τ_SR_ = 0.05–0.025 a.u. are used in the SR method during VMC optimization. A low-memory conjugate-gradient algorithm^60^ is used to solve for the wave function parameters in the SR equations. All DMC calculations use a time step of τ_DMC_ = 0.02 a.u.

A value of λ_*IJ*_ = 1.0 a.u. is used for all two-state calculations, while multiple values are used for the three-state system, as discussed in the previous section and in the Supporting Information.

## 4 Results

### 4.1 Nitroxyl 1^1^A′ → 2^1^A′ Double Excitation

This transition in nitroxyl is a pure double (*n*, *n*) → (π*, π*) excitation. The VMC and DMC energies and VTEs are shown in Table 1. There are reliable benchmark calculations for the 1^1^A′ → 2^1^A′ VTE in nitroxyl. In particular, the exFCI/aug-cc-pVQZ (AVQZ) calculation predicts the lowest value for this excitation at 4.32 eV. The latest^16^ CC4 and CCSDTQ calculations using the aug-cc-pVTZ (AVTZ) basis set somewhat overestimate it at 4.380 and 4.364 eV, respectively.

**Table 1:** Nitroxyl VMC and DMC Total Energies (a.u.) and Excitation Energies (eV)^a^

			VMC			DMC		
WF	# det	# parm	*E*(1^1^A′)	*E*(2^1^A′)	Δ*E*	*E*(1^1^A′)	*E*(2^1^A′)	Δ*E*
CIPSI	321	850	–26.4917(2)	–26.3322(2)	4.34(1)	–26.5164(2)	–26.3583(2)	4.30(1)
	1573	1446	–26.5001(2)	–26.3415(2)	4.31(1)	–26.5220(2)	–26.3636(2)	4.31(1)
	2900	2002	–26.5040(2)	–26.3447(2)	4.34(1)	–26.5240(2)	–26.3652(2)	4.32(1)
	7171	4124	–26.5102(2)	–26.3498(2)	4.36(1)	–26.5267(2)	–26.3677(2)	4.33(1)
	10,690	5712	–26.5113(2)	–26.3519(2)	4.34(1)	–26.5273(2)	–26.3686(2)	4.32(1)
TBE^b^								4.33
CC3/AVQZ^c^								5.23
CC4/AVDZ^d^								4.454
CC4/AVTZ^d^								4.380
CCSDT/AVDZ^c^								4.756
CCSDTQ/AVDZ^c^								4.424
CCSDTQ/AVTZ^d^								4.364
exFCI/AVQZ^e^								4.32(0)
CASPT2(12,9)/AVQZ^e^								4.34
PC-NEVPT2(12,9)/AVQZ^e^								4.35

a The number of determinants (# det) and parameters (# parm) in each trial wave function are also listed. The CIPSI expansions are performed on a NO basis and fully reoptimized in VMC.

b This value is “safe”, as defined in ref (12), FCI/AVTZ.

c Ref (15).

d Ref (16).

e Ref (13).

**Figure 3:**
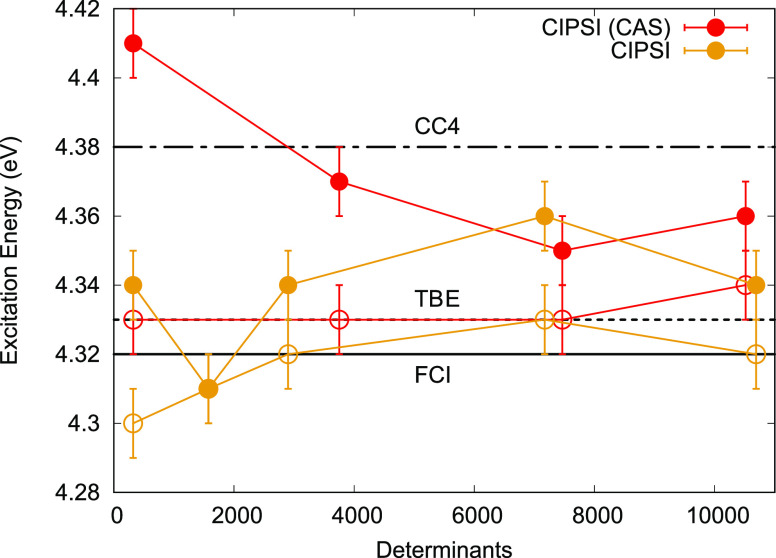
Nitroxyl VMC (filled points) and DMC (empty points) excitation energies for different CIPSI wave functions expanded either on starting CAS [SA(2)-CASSCF(12,9)] or NO basis and fully reoptimized in VMC. Horizontal lines represent reference values. TBE: FCI/ATVZ, FCI: exFCI/AVQZ, and CC4: CC4/AVTZ.

CIPSI wave functions are expanded in a CAS orbital basis [SA(2)-CASSCF(12,9)] as well as in an NO basis while matching the PT2 energies and variance. Upon state-specific optimization of the Jastrow-CIPSI wave functions, the VMC and DMC VTEs are very similar for different matching and orbital basis (see Figure 3 and Supporting Information), and all are in very good agreement with benchmarks. In addition, consistency of VMC with the DMC excitation energy is reached at fairly small determinant expansions (∼2000). Due to the fast convergence of the VMC and DMC excitation energies, the use of NOs in the CIPSI expansion is not necessary but still provides similar results.

Previous results for nitroxyl show that at least CC4 and CCSDTQ are necessary to capture the important correlations of the 1^1^A′ → 2^1^A′ double excitation. There is also a strong basis set dependence on the excitation with CC methods, yet the VMC and DMC calculations, starting from AVDZ with pseudopotentials, reach agreement with expensive quantum chemistry calculations which require the use of at least AVTZ basis set.

### 4.2 Glyoxal 1^1^A_g_ → 2^1^A_g_ Double Excitation

The pure double excitation in glyoxal also corresponds to a (*n*, *n*) → (π*, π*) transition. Since the system size is larger compared to nitroxyl, the same level of CC4 and CCSDTQ calculations is not available, and there appears to be a large basis set dependence on the most recent CC4 data.^16^ A symmetry adapted cluster CI (SAC-CI) calculation of this excitation is available^61^ (5.66 eV) and agrees well with the recent TBE, which is a basis set corrected FCI/AVDZ value of 5.61 eV.^12^ These values also agree well with the highest level CCSDTQ estimate for this VTE, 5.670 eV,^16^ all shown and compared to our QMC results in Table 2.

**Table 2:** Glyoxal VMC and DMC Total Energies (a.u.) and Excitation Energies (eV)^a^

			VMC			DMC		
WF	# det	# parm	*E*(1^1^A_g_)	*E*(2^1^A_g_)	Δ*E*	*E*(1^1^A_g_)	*E*(2^1^A_g_)	Δ*E*
CIPSI	3749	2908	–44.6072(2)	–44.3957(2)	5.76(1)	–44.6564(2)	–44.4482(2)	5.67(1)
	5046	3498	–44.6127(2)	–44.4004(2)	5.78(1)	–44.6600(2)	–44.4509(2)	5.69(1)
	7040	4500	–44.6150(2)	–44.4048(2)	5.72(1)	–44.6613(2)	–44.4534(2)	5.66(1)
	8642	5433	–44.6181(2)	–44.4098(2)	5.67(1)	–44.6632(2)	–44.4559(2)	5.64(1)
	10,010	6188	–44.6205(2)	–44.4114(2)	5.69(1)	–44.6635(2)	–44.4567(2)	5.63(1)
	12,132	6269	–44.6237(2)	–44.4145(2)	5.70(1)	–44.6654(2)	–44.4576(2)	5.66(1)
	15,349	8577	–44.6276(2)	–44.4180(2)	5.70(1)	–44.6670(2)	–44.4600(2)	5.63(1)
TBE^b^								5.61
CC3/AVQZ^c^								6.76
CC4/6-31+G*^d^								5.699
CC4/AVDZ^d^								5.593
CCSDTQ/6-31+G*^d^								5.670
exFCI/AVDZ								5.56(11)
SAC-CI/AVDZ^e^								5.66
CASPT2(14,12)/AVQZ^c^								5.43
PC-NEVPT2(14,12)/AVQZ^c^								5.52

a The number of determinants (# det) and parameters (# parm) in each trial wave function are also presented. The CIPSI expansions are performed on a NO basis and fully reoptimized in VMC.

b This value is “safe”, as defined in ref (12), exFCI/AVDZ + (CCSDT/AVTZ-CCSDT/AVDZ).

c Ref (13).

d Ref (16).

e Ref (61), see reference for basis set details.

**Figure 4:**
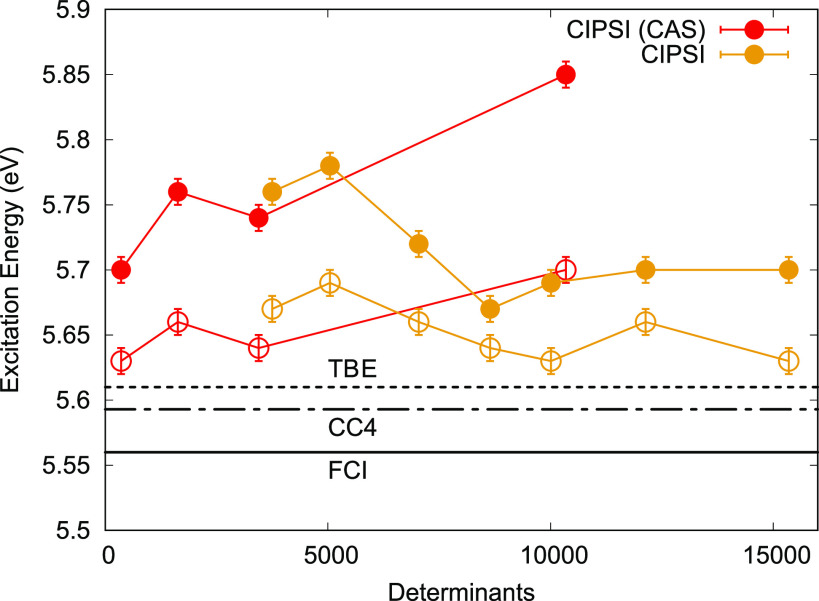
Glyoxal VMC (filled circles) and DMC (empty circles) excitations energies for different CIPSI wave functions expanded either on starting CAS [SA(2)-CASSCF(14,12)] or NO basis and fully reoptimized in VMC. Horizontal lines represent reference values. TBE: FCI/AVDZ + (CCSDT/AVTZ – CCSDT/AVDZ), CC4: CC4/AVDZ, and FCI: exFCI/AVDZ (error bar of ±0.11 eV).

PT2 and variance-matched CIPSI expansions are generated from the optimal orbitals of a SA(2)-CASSCF(14,12) calculation. As shown in Figure 4, after the optimization of the CIPSI (CAS) wave functions, we observe an increase in the VMC excitation energy as the expansion grows from about 400 to 10,000 determinants. The use of DMC mitigates this increase. The increasing excitation energy is related to the poorer matching at 10,000 determinants than that at smaller expansions for the chosen fixed weights in the CIPSI calculations and is also mirrored in the behavior of the corresponding CIPSI excitations. Using an automated matching algorithm like for cyclopentadienone would resolve the problem (see also the Supporting Information for QMC calculations for glyoxal with different weights). The use of a single set of weights is not an issue for the glyoxal CIPSIs in an NO basis.

When using NOs built from modest-sized CIPSI expansions (10^4^ determinants) and reperforming the CIPSI expansions, in the presence of the Jastrow factor, the VMC and DMC excitation energies appear to converge nicely and to a value in agreement with available benchmarks. It is possible that still larger CIPSI expansions and more rounds of NO generation could bring the VMC into better alignment with the DMC, which differs at most by 0.05 eV. The DMC value for the largest determinant expansion in the NO basis is 5.63(1) eV, which is ∼0.01 eV from the current TBE value and ∼0.02 eV from the SAC-CI/AVDZ and CC4/AVDZ values. This close agreement and the consistency of the DMC results with different number of determinants in the CIPSI wave functions suggest the value of 5.63(1) eV to be a reasonable prediction for this excitation.

### 4.3 Tetrazine 1^1^A_1g_ → 2^1^A_1g_ Double Excitation

The tetrazine (*s*-tetrazine) pure double excitation of interest is another (*n*, *n*) → (π*, π*) transition. There are few reliable benchmark calculations available for this transition due to the molecule’s size in addition to its genuine double nature. Although CC3 does not properly treat double excitations, calculations using 6-31+G* up to AVQZ show that there is very little basis set effect, only a change of ∼0.03 eV on the excitation energy. If the CC4/6-31+G* value of 5.06 eV is just as similar for larger basis sets, then this value can be seen as a good guess for the VTE. In any case, without the calculations available to be sure, the VMC and DMC presented in Table 3 suggest a value close to 5.0 eV for the VTE.

**Table 3:** Tetrazine VMC and DMC Total Energies (a.u.) and Excitation Energies (eV)^a^

			VMC			DMC		
WF	# det	# parm	*E*(1^1^A_1g_)	*E*(2^1^A_1g_)	Δ*E*	*E*(1^1^A_1g_)	*E*(2^1^A_1g_)	Δ*E*
CIPSI	3572	2414	–52.2198(2)	–52.0346(2)	5.04(1)	–52.2935(3)	–52.1098(3)	5.00(1)
	7025	4141	–52.2300(2)	–52.0451(2)	5.03(1)	–52.2981(3)	–52.1148(3)	4.99(1)
	10,723	6053	–52.2379(2)	–52.0528(2)	5.04(1)	–52.3015(3)	–52.1182(3)	4.99(1)
TBE^b^								4.61
CC3/6-31+G*^c^								6.22
CC3/AVQZ^c^								6.19
CC4/6-31+G*^d^								5.06
CCSDT/AVTZ^c^								5.96
exFCI/AVDZ								5.15(3)
CASPT2(14,10)/AVQZ^c^								4.68
PC-NEVPT2(14,10)/AVQZ^c^								4.60

a The number of determinants (# det) and parameters (# parm) in each trial wave function are also presented. The CIPSI expansions are performed on a NO basis and fully optimized in VMC.

b This value is “unsafe,” as defined in ref (12), NEVTPT2/AVTZ.

c Ref (13).

d Ref (16).

**Figure 5:**
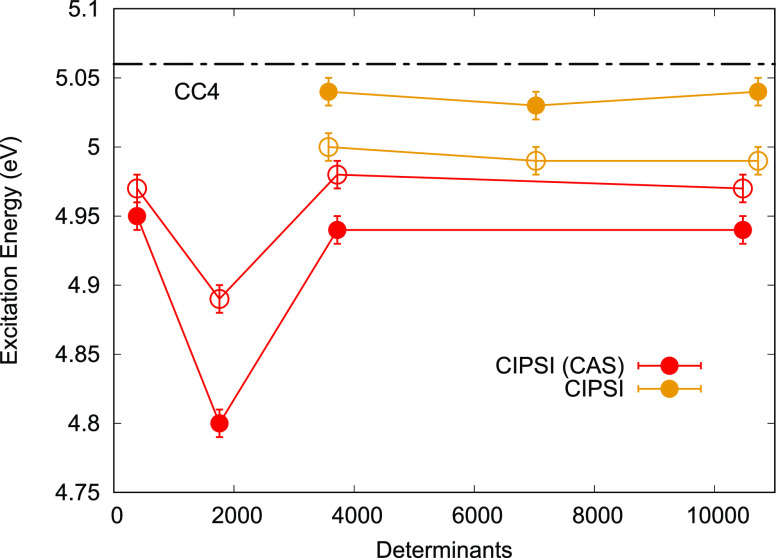
Tetrazine VMC (filled circles) and DMC (empty circles) excitation energies for different CIPSI wave functions expanded either on starting CAS [SA(2)-CASSCF(14,10)] or NO basis and fully reoptimized in VMC. Horizontal lines represent the reference value (CC4: CC4/6-31+G*).

The VMC optimization of the CAS orbital-based [SA(2)-CASSCF(14,10)] CIPSI expansions tends toward 4.94(1) eV (see the Supporting Information) while the NO-based CIPSI goes to 5.04(1) eV, both converging after about 3600–3700 determinants. While the converged VMC excitation energies are not quite in agreement, their DMC values are consistent, with VTEs of 4.97(1) and 4.99(1) eV at the largest determinant expansions (see Figure 5). Even more than in glyoxal, the consistency of the DMC value for the CIPSI expansions of different lengths and initial MO basis sets suggests that it should be a trusted benchmark for this transition.

### 4.4 3-State VMC Optimization: Cyclopentadienone 1^1^A_1_ → 2^1^A_1_ and 1^1^A_1_ → 3^1^A_1_ Excitations

To show the capabilities of the VMC optimization method, the three lowest ^1^A_1_ states of cyclopentadienone are optimized simultaneously to determine the two lowest excitation energies. One is deemed a double excitation (π, π → π*, π*) with ∼50% *T*_1_ and the other a single excitation (π → π*) with ∼74% *T*_1_ by CC3 calculations. All results are collected in Table 4.

**Table 4:** Cyclopentadienone VMC and DMC Excitation Energies (eV)^a^

			VMC			DMC		
WF	# det	# parm	Δ*E*_12_	Δ*E*_13_	Δ*E*_23_	Δ*E*_12_	Δ*E*_13_	Δ*E*_23_
CIPSI	1025	2403	5.94(1)	6.92(1)	0.98(1)	5.90(1)	6.87(1)	0.97(1)
	3033	3645	5.95(1)	6.96(1)	1.01(1)	5.87(1)	6.87(1)	1.00(1)
	7151	5365	6.03(1)	7.05(1)	1.02(1)	5.92(1)	6.91(1)	0.99(1)
	10,206	6574	6.04(1)	7.03(1)	0.99(1)	5.90(1)	6.89(1)	0.99(1)
TBE^b^						6.00^c^	6.09^d^	0.09
ADC(3)/AVTZ						4.59^e^	6.50^e^	1.91
CC2/AVTZ							6.50^e^	
CC3/AVTZ						7.10^e^	6.21^e^	–0.89
CCSD/AVTZ							6.68^e^	
CCSDT-3/AVTZ							6.33^e^	
exFCI/AVDZ						5.81(39)	6.93(24)	1.10(13)

a The number of determinants (# det) and parameters (# parm) in each trial wave function is also listed. The CIPSI expansions are performed on a NO basis and fully optimized in VMC. Reference values for the double and single excitation are placed in the Δ*E*_12_ and Δ*E*_13_ columns, respectively, to match the order of the states calculated with VMC and DMC. The VMC optimization of the two smaller CIPSIs used λ_*IJ*_ = (1.5, 2.0, 1.0) and, of the two larger, (1.0, 1.0, 1.0).

b This value is “unsafe,” as defined in ref (12).

c NEVPT2/AVTZ.

d CCSDT/AVDZ + (CC3/AVTZ – CC3/AVDZ).

e These values are also “unsafe”.

CC3/AVTZ predicts a value of 7.10 eV for the double excitation and 6.21 eV for the single excitation, which is the opposite of what is found here. The CC3 value for the double excitation is not trustable since it misses quadruple excitations important for describing this type of excitation. Véril *et al.*(12) determine the CC3 and CCSDT calculations of these excitations to be “unsafe” since they do not agree to within 0.03–0.04 eV. The relatively low *T*_1_ of the single excitation could lead to a poor treatment by CC3 and CCSDT. No CC4 or CCSDTQ calculations are currently available for reliable comparison to the present results for the double excitation energy, and all benchmark VTEs in Table 4 are considered unsafe. An exFCI/AVDZ calculation is performed in the present work, although it has fairly large error bars.

**Figure 6:**
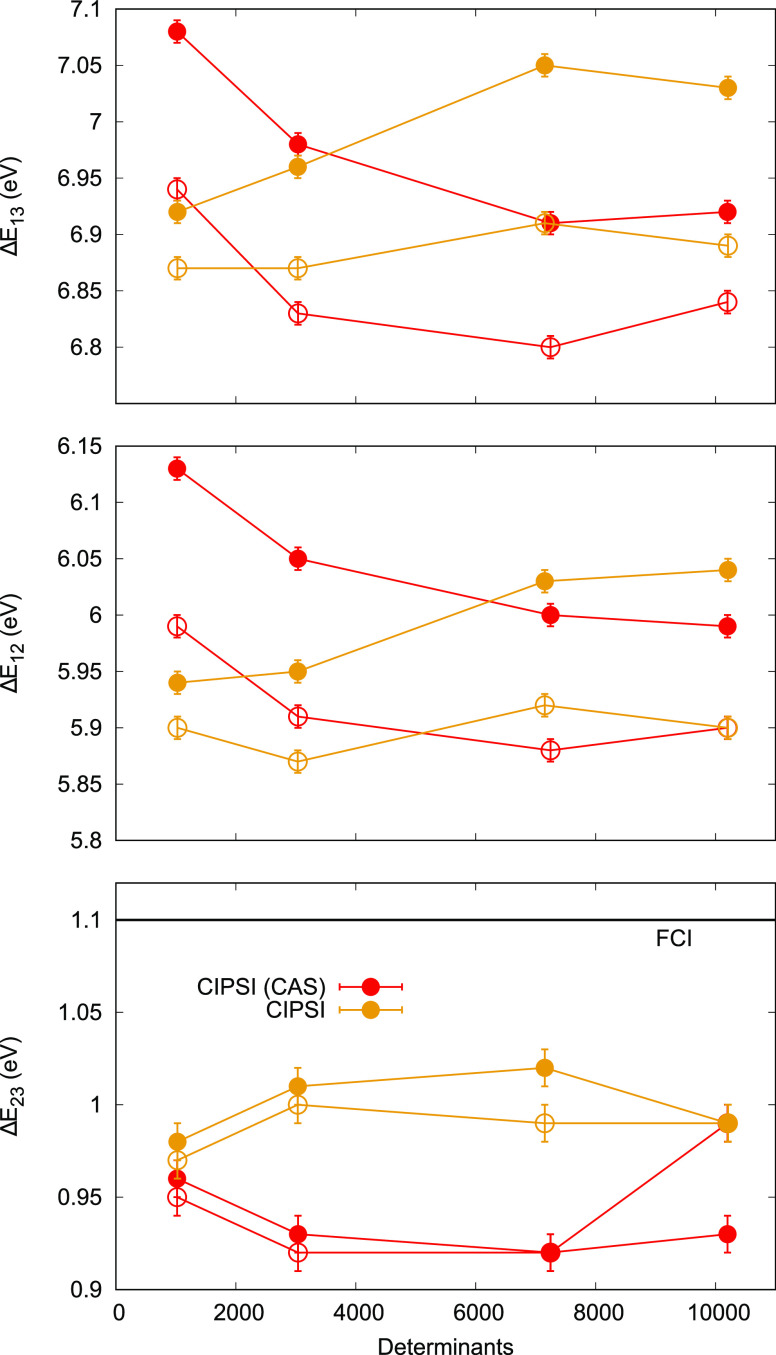
Cyclopentadienone VMC (filled circles) and DMC (empty circles) excitation energies for different CIPSI wave functions expanded either on starting CAS [SA(3)-CASSCF(6,6)] or NO basis and fully optimized in VMC. FCI: exFCI/AVDZ (error bar of ±0.13 eV applies to the FCI value for Δ*E*_23_).

All VMC and DMC performed on CIPSI wave functions give roughly the same results (see Table 4, Figure 6, and Supporting Information). The largest CIPSI wave function in the NO basis has DMC Δ*E*_12_, Δ*E*_13_, and Δ*E*_23_ values of 5.90(1), 6.89(1), and 0.99 eV, respectively. We find that the two smaller determinant expansions in Table 4 require larger values of λ_*IJ*_ to stabilize the overlaps (see also the Supporting Information for further discussion).

The present calculations definitively show the ordering of the two lowest ^1^A_1_ excited states. The largest CIPSI calculation performed in the CAS orbital basis [SA(3)-CASSCF(6,6)] finds the 2^1^A_1_ state to have coefficients of 0.70 and 0.46 for the dominant doubly [(π, π) → (π*, π*)] and singly excited (π → π*) CSFs, respectively, while the 3^1^A_1_ state has coefficients of 0.49 and 0.75. The coefficients show a clear mixing of singly and doubly excited determinants in both states (also suggested by the *T*_1_ values), but the 2^1^A_1_ state is clearly distinguishable as the double excitation and the 3^1^A_1_ state as the single.

## 5 Conclusions

With the use of a penalty-based, state-specific optimization scheme, we show that the same QMC method which provides accurate single-excitations energies can also be applied to double excitations. That is, we use PT2 energy and variance-matched CIPSI determinant expansions as the Slater part of the Jastrow–Slater trial function in the VMC optimization. The introduction of an objective function allows a state-specific optimization of multiple states of the same symmetry simultaneously by imposing orthogonality between all eigenstates.

For optimization with two states, it is sufficient to apply a large enough λ_12_ to stabilize the optimization. With more states, there are wide ranges of the various λ_*IJ*_ which give consistent and stable excitation energies. Good values can be found at a preliminary stage with relatively few iterations.

The pure double excitations in nitroxyl [(4.32(1) eV] and glyoxal [5.63(1) eV] calculated here are in excellent agreement with reliable benchmarks in the literature. We also expect that our VTEs calculated at the level of DMC are reliable benchmarks for molecules and excitation types where CC methods are too costly to do with a large enough basis set. Specifically, we suspect a CC4 calculation with a larger basis set will find a tetrazine double excitation close to our result, 4.99(1) eV, slightly lower than the CC4/6-31G* result. For cyclopentadienone, the ^1^A_1_ first excited state (2^1^A_1_) is predominantly a double excitation at 5.90(1) eV, while the second excited state is predominantly a single excitation at 6.89(1) eV.

The favorable computational scaling of QMC with the system size and its alignment with modern supercomputers make it a serious candidate for applications involving complex excited states, such as long-range charge-transfer excitations, conical intersections, or problems where the ordering between excited states of different characters is unclear.

## Supporting Information Available

Choice of input weights for the matching of multiple CIPSI wave functions; CIPSI energies, PT2 energies, and variances for the expansions used in the QMC calculations; additional QMC total and excitation energies obtained with CAS wave functions and with CIPSI determinant components originally expressed in a CAS orbital basis; additional two- and three-state optimization calculations for nitroxyl; and overlap data for cyclopentadienone and different wave functions (PDF)
