# Supplementary material for: Double Excitation Energies from Quantum Monte Carlo Using State-Specific Energy Optimization
Source: J Chem Theory Comput. 2022 Oct 31;18(11):6722–31. doi: 10.1021/acs.jctc.2c00769 (PMC9648196; doi:10.1021/acs.jctc.2c00769)
Supplement: Supplementary file 1 — ct2c00769_si_001.pdf [file ct2c00769_si_001.pdf]

# Supporting Information: Double excitation energies from quantum Monte Carlo using state-specific energy optimization

Stuart Shepard,[*,†] Ramón L. Panadés-Barrueta,[†,§] Saverio Moroni,[*,‡] Anthony Scemama,[*,¶] and Claudia Filippi[*,†]

†*MESA+ Institute for Nanotechnology, University of Twente, 7500 AE Enschede, The Netherlands*

‡*CNR-IOM DEMOCRITOS, Istituto Officina dei Materiali and SISSA Scuola Internazionale Superiore di Studi Avanzati, Via Bonomea 265, I-34136 Trieste, Italy*

¶*Laboratoire de Chimie et Physique Quantiques, Université de Toulouse, CNRS, UPS, 31062 Toulouse, France*

§*Current address: Faculty of Chemistry and Food Chemistry, Technische Universität Dresden, 01062 Dresden, Germany*

E-mail: s.s.shepard@utwente.nl; moroni@democritos.it; scemama@irsamc.ups-tlse.fr; c.filippi@utwente.nl

# 1 CASSCF Calculations

All SA($N_{\mathrm{states}}$)-CASSCF($N_{\mathrm{e}}$,$M_{\mathrm{orb}}$) calculations are performed in GAMESS(US)[1] using BFD pseudopotentials with the corresponding aug-cc-pVDZ basis set. All geometries used are from ground-state optimizations performed at the level of CC3/aug-cc-pVTZ.[2,3].

All states of interest belong to the same irreducible representation as the ground state, so they have the fully-symmetric representation of the molecular geometry point-group symmetry. The point group symmetries used for nitroxyl, glyoxal, s-tetrazine, and cyclopentadienone are $\mathrm{C_s}$, $\mathrm{C_{2h}}$, $\mathrm{D_{2h}}$, and $\mathrm{C_{2v}}$, respectively.

For all molecules, we report below the sizes of the CAS used, and the total and excitation energies obtained at the CASSCF level.

## 1.1 Nitroxyl

Table 1: Summary of SA(2)-CASSCF($N_{\mathrm{e}}$,$M_{\mathrm{orb}}$) calculations on nitroxyl. The CASSCF energies ($E$) and excitation energies ($\Delta E$) are given in Hartree (H) and eV, respectively. All reference calculations are all-electron SA(2)-CASSCF calculations.

| | # CSFs | $E(1^1\mathrm{A}')$ | $E(2^1\mathrm{A}')$ | $\Delta E$ |
|---|---|---|---|---|
| CAS(2,2) | 2 | -25.997981 | -25.823970 | 4.74 |
| CAS(12,9) | 1316 | -26.127782 | -25.955779 | 4.68 |
| CAS(12,9)/AVDZ[a] | | -129.932723 | -129.760088 | 4.70 |
| CAS(12,9)/AVQZ[a] | | -129.969310 | -129.797750 | 4.67 |

[a]Ref. 3

## 1.2 Glyoxal

Table 2: Summary of SA(2)-CASSCF($N_e$,$M_{orb}$) calculations on glyoxal. The CASSCF energies ($E$) and excitation energies ($\Delta E$) are given in Hartree (H) and eV, respectively. All reference calculations are all-electron SA(2)-CASSCF calculations.

| | # CSFs | $E(1^1\mathrm{A_g})$ | $E(2^1\mathrm{A_g})$ | $\Delta E$ |
|---|---|---|---|---|
| CAS(4,4) | 8 | -43.735960 | -43.568786 | 4.55 |
| CAS(8,6) | 33 | -43.810454 | -43.597550 | 5.79 |
| CAS(14,12) | 42756 | -43.922989 | -43.705180 | 5.93 |
| CAS(8,6)/AVDZ$^a$ | | -226.685161 | -226.471894 | 5.80 |
| CAS(8,6)/AVQZ$^a$ | | -226.752190 | -226.540026 | 5.77 |
| CAS(14,12)/AVDZ$^a$ | | -226.794485 | -226.576274 | 5.94 |
| CAS(14,12)/AVQZ$^a$ | | -226.862224 | -226.644714 | 5.92 |

$^a$Ref. 3

## 1.3 Tetrazine

Table 3: Summary of SA(2)-CASSCF($N_e$,$M_{orb}$) calculations on tetrazine. The MCSCF energies ($E$) and excitation energies ($\Delta E$) are given in Hartee (H) and eV, respectively. All reference calculations are all-electron SA(2)-CASSCF calculations.

| | # CSFs | $E(1^1\mathrm{A_{1g}})$ | $E(2^1\mathrm{A_{1g}})$ | $\Delta E$ |
|---|---|---|---|---|
| CAS(4,4) | 6 | -51.028815 | -50.806971 | 6.04 |
| CAS(8,6) | 21 | -51.079408 | -50.821626 | 7.02 |
| CAS(12,8) | 56 | -51.079992 | -50.846778 | 6.35 |
| CAS(14,10) | 670 | -51.130154 | -50.930008 | 5.45 |
| CAS(14,10)/AVDZ$^a$ | | -294.727767 | -294.526292 | 5.48 |
| CAS(14,10)/AVQZ$^a$ | | -294.803516 | -294.604540 | 5.41 |

$^a$Ref. 3

## 1.4 Cyclopentadienone

Table 4: Summary of SA($N_{\rm state}$)-CASSCF($N_{\rm e}$, $M_{\rm orb}$) calculations on cyclopentadienone. The CASSCF energies ($E$) and excitation energies ($\Delta E$) are given in Hartree (H) and eV, respectively.

| | # CSFs | $E(1^1\mathrm{A}_1)$ | $E(2^1\mathrm{A}_1)$ | $E(3^1\mathrm{A}_1)$ | $\Delta E_{12}$ | $\Delta E_{13}$ | $\Delta E_{23}$ |
|---|---|---|---|---|---|---|---|
| SA(3) | | | | | | | |
| CAS(6,6) | 95 | -45.749640 | -45.519741 | -45.437557 | 6.26 | 8.49 | 2.24 |
| CAS(8,7) | 152 | -45.749865 | -45.519864 | -45.437841 | 6.26 | 8.49 | 2.23 |
| CAS(10,8) | 326 | -45.752040 | -45.521488 | -45.438852 | 6.27 | 8.52 | 2.25 |
| SA(4) | | | | | | | |
| CAS(6,6) | 95 | -45.747679 | -45.520726 | -45.434884 | 6.18 | 8.51 | 2.34 |
| CAS(8,7) | 152 | -45.747963 | -45.520831 | -45.435133 | 6.18 | 8.51 | 2.33 |
| CAS(10,8) | 326 | -45.750131 | -45.522663 | -45.435928 | 6.19 | 8.55 | 2.36 |
| SA(5) | | | | | | | |
| CAS(6,6) | 95 | -45.744381 | -45.518430 | -45.432765 | 6.15 | 8.48 | 2.33 |
| CAS(8,7) | 152 | -45.747246 | -45.518198 | -45.435403 | 6.23 | 8.49 | 2.25 |
| CAS(10,8) | 326 | -45.748150 | -45.519019 | -45.436202 | 6.23 | 8.49 | 2.25 |

# 2 CIPSI calculations

## 2.1 PT2 and Variance Matching

At each iteration in the CIPSI algorithm, a new set of determinants is added to the determinant space, shared by all states. In Quantum Package[4,5] (QP), one controls how many determinants are added at each iteration via the selection factor, $f$, given by,

$$N_\alpha = \mathrm{INT}(\sqrt{N_{\mathrm{states}}} \times N_{\mathrm{det}} \times f), \tag{1}$$

where $N_\alpha$ is the number of new determinants to add to the expansion and $N_{\mathrm{det}}$ is the number determinants currently in the expansion.

The wave functions of the different states are said to be 'matched' when they have similar PT2 energies ($E_{\mathrm{PT2}}$) and variances ($\sigma^2$), which quantify the quality of the wave function. In QP, the matching is controlled at each iteration by calculating selection weights on each state, $W_I$, which then bias the selection of determinants for the improvement of a given state. An external determinant $|\alpha\rangle$ is selected based on its weighted PT2 energy contribution obtained via the Epstein-Nesbet partitioning of the Hamiltonian,

$$e_\alpha = \sum_{I=1}^{N_{\mathrm{states}}} W_I \delta E^{(2)}_{\alpha,I}, \tag{2}$$

where

$$\delta E^{(2)}_{\alpha,I} = \frac{|\langle \alpha|H|\Psi_I^{\mathrm{CIPSI}}\rangle|^2}{\langle \Psi_I^{\mathrm{CIPSI}}|H|\Psi_I^{\mathrm{CIPSI}}\rangle - \langle \alpha|H|\alpha\rangle}\,, \tag{3}$$

or based on its weighted contribution to the variance of the states

$$\sigma^2_\alpha = \sum_{I=1}^{N_{\mathrm{states}}} W_I \delta\sigma^2_{\alpha,I}, \tag{4}$$

where

$$\delta\sigma^2_{\alpha,I} = |\langle\alpha|H|\Psi_I^{\mathrm{CIPSI}}\rangle|^2, \tag{5}$$

and $\Psi_I^{\mathrm{CIPSI}}$ is the current normalized CIPSI wave function for state $I$. At a given iteration, the larger the selection weight for a state, the more likely determinants will be added to the expansion which benefit that state. The goal is to keep the quality of the wave functions similar during the expansion.

The simplest way to calculate $W_I$ is by using the largest expansion coefficient in the current wave function for each state, ${}^{i}c_I^{\max}$, where $i$ is the current iteration. The iteration index will be removed until a distinction between iterations must be made. The unnormalized weight is given by, $\tilde{w}_I^{\mathrm{c_{max}}} = 1/|c_I^{\max}|^2$. The normalized weights are given by

$$w_I^{\mathrm{c_{max}}} = \frac{\tilde{w}_I^{\mathrm{c_{max}}}}{\sum_I^{N_{\mathrm{states}}} \tilde{w}_I^{\mathrm{c_{max}}}}. \tag{6}$$

Two different approaches are used to calculate $W_I$, both of which include the weights in Eq. 6.

In the first approach (i), additional input weights, $w_I^{\mathrm{inp}}$, (which sum to unity) are chosen by hand and they remain fixed throughout the calculation[6]. These are the same weights denoted as $w_I^{\mathrm{SA}}$ in Ref.[6], but we wish to emphasize here that these weights are provided as input. The input weights enter the selection weights at each iteration as

$$\tilde{W}_I = (w_I^{\mathrm{c_{max}}}) \times (w_I^{\mathrm{inp}}), \tag{7}$$

where a tilde symbol indicates that the weight is not normalized.

In the second approach (ii), weights are constructed from the PT2 energies, $w_I^{\mathrm{PT2}}$, and variance, $w_I^{\sigma^2}$, and are used to calculate the selection weights. These two unnormalized weights are calculated according to

$${}^{i}\tilde{w}_I^{\mathrm{PT2}} = ({}^{i-1}w_I^{\mathrm{PT2}}) \times ({}^{i}E_I^{\mathrm{PT2}}), \tag{8}$$

$${}^{i}\tilde{w}_I^{\sigma^2} = ({}^{i-1}w_I^{\sigma^2}). \times ({}^{i}\sigma_I^2) \tag{9}$$

Note that the unnormalized weights at the present iteration, $i$, are calculated using the normalized weights from the previous iteration, which are set to unity at the start of the calculation. These weights do not affect the selection weight until 100 determinants have been reached. After the weights in Eqs. 8 and 9 are normalized in the same way as in Eq. 6, the unnormalized selection weights for approach (ii) are calculated as

$$\tilde{W}_I = (w_I^{c_{\max}}) \times \sqrt{(w_I^{\mathrm{PT2}}) \times (w_I^{\sigma^2})}. \tag{10}$$

Therefore, the selection weight is larger for a state with smaller $c_I^{\max}$, but larger $E_I^{\mathrm{PT2}}$ and $\sigma_I^2$, maintaining wave functions of similar quality.

Approach (i) is used for all molecules, while (ii) is only utilized for cyclopentadienone. Using different input weights in approach (i) will lead to a different evolution of the determinant expansion, and a slightly different number of determinants added at each CIPSI iteration. Therefore, to compare expansions with different input weights in Section 3, expansions with similar number of determinants are provided as best as possible. The optimal CIPSI input weights, $w_I^{\mathrm{inp}}$, for different systems together with the starting orbitals and expansion are given in the next Section.

## 2.2 Starting Wave Functions

In a multi-state CIPSI calculation it is important to make sure the correct excited state(s) are found early in the CIPSI expansion. In general, performing a CI including singles and doubles (CISD) calculation ensures the lowest energy states in a given symmetry are found. The CISD can be performed iteratively in QP and stopped after reaching a predetermined number of determinants, $N_{\mathrm{det}}^{\max}$.

For nitroxyl, glyoxal, and tetrazine, the excitations of interest are dominated by closed-shell doubly-excited determinants, so it is more appropriate to perform a seniority-zero, doubly occupied CI (DOCI) to build the initial CIPSI wave function. This determinant expansion is then truncated to include the determinants with largest expansion coefficients (between 2 and 51 determinants).

This initial wave function ensures the double excitation of interest is found immediately in the CIPSI calculation. Starting from this wave function, an unrestricted CIPSI is performed up to about $10^4$ determinants.

For cyclopentadienone, the excited-states of interest have a large mix of singly- and doubly-excited determinants so an iterative CISD up to $\sim 10^3$ determinants is performed and then truncated to $\sim 100$ determinants to initialize the wave function.

It is found, in general, that as long as the dominant determinant is included in the starting wave function the CIPSI expansion will follow a similar evolution. This is made clear by Figure 1 below. The CIPSI performed on glyoxal starting from a DOCI truncated to 2 determinants (DOCI[HF+1]) versus 51 determinants (DOCI[HF+50]) made little difference in the evolution of the energies beyond 50 determinants. Data on all starting CIPSI wave functions are provided in Table 5.

Table 5: Summary of CIPSI starting wave functions (WF), orbitals, and optimal weights ($w_I^{\text{inp}}$) providing the best PT2 and variance matching. For the NO orbitals, we denote with *method*(*HF+# dets after truncation*)*-# dets in CIPSI* the wave function from which the NO orbitals are calculated: for instance, DOCI(HF+1)-10000 means that the NO orbitals are calculated from a first CIPSI expansion to about 10000 determinants, starting from a DOCI truncated to 2 determinants. Note that the matching algorithm (ii) described in the previous Section is used for cyclopentadienone in the NO basis.

| | CAS | | | NO | | |
|---|---|---|---|---|---|---|
| Molecule | Orbitals | WF | $w_I^{\text{inp}}$ | Orbitals | WF | $w_I^{\text{inp}}$ |
| Nitroxyl | SA(2)-CASSCF(12,9) | DOCI(HF+1) | 0.525−0.475 | DOCI(HF+1)-10000 | DOCI(HF+1) | 0.525−0.475 |
| Glyoxal | SA(2)-CASSCF(14,12) | DOCI(HF+1) | 0.50−0.50 | DOCI(HF+50)-10000 | DOCI(HF+50) | 0.45−0.55 |
| Tetrazine | SA(2)-CASSCF(14,10) | DOCI(HF+1) | 0.45−0.55 | DOCI(HF+50)-10000 | DOCI(HF+50) | 0.45−0.55 |
| Cyclopentadienone | SA(3)-CASSCF(6,6) | CISD(HF+97) | 0.27−0.35−0.38 | CISD(HF+97)-20000 | CISD(HF+101) | − |

## 2.3 Molecular Orbital Basis

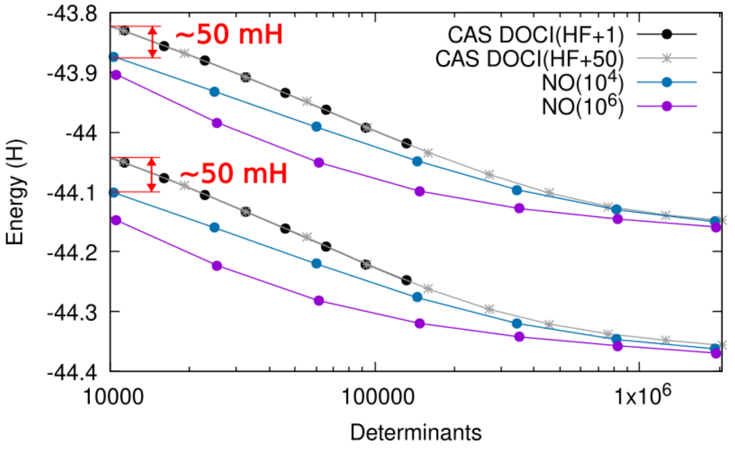


Figure 1: Glyoxal ground- and excited-state CIPSI energies using different starting wave functions and MO basis. The two CIPSI expansions performed in the CAS basis show that different starting wave functions lead to the same evolution of the energies. The two CIPSI expansions performed in the NO basis both start from a DOCI(HF+50) wave function, but one set of NOs were generated from a previous CIPSI wave function with $10^4$ determinants and the other with $10^6$. The same input weights ($w_1^{\text{inp}}$ $w_2^{\text{inp}}$ : 0.5 0.5) and basis set are used in all calculations.

The CIPSI expansions are performed in a molecular orbital (MO) basis built from either the CASSCF optimized orbitals of a SA($N_{\text{states}}$)-CASSCF($N_{\text{e}}$,$M_{\text{orb}}$) calculation (referred to as CAS) or the natural orbitals (NO) built from a CIPSI expansion performed in the CAS basis.

The choice of MO basis (CAS versus NO) has a noticeable effect on the energies in the CIPSI expansion. Figure 1 illustrates how the ground- and excited-state energies of glyoxal are affected when choosing different starting wave functions and size of the CIPSI used to build the NOs. The energies of the ground- and excited-states all tend towards the same energy as the expansion reaches $10^6$ determinants, but the energies around $10^4$ determinants are noticeably different for different MO basis. Using NOs from a $10^4$ and a $10^6$ determinant expansion lowers the energy of both states by $\sim$50 mH and $\sim$70 mH, respectively, compared to the CAS basis. This suggests a better quality wave function is obtained in the NO basis for the same size determinant expansions. This is also expected to lead to a faster convergence of the VMC/DMC VTEs at smaller determinant expansion. Details on the MO basis for all CIPSI calculations are provided in Table 5.

# 3 CIPSI Data

In the following, relevant data from CIPSI calculations are provided, including CIPSI energies ($E_{\mathrm{CIPSI}}$), PT2 energies ($E_{\mathrm{PT2}}$), and variances ($\sigma^2$) for ground and excited states. The differences are also provided and are always calculated as $\Delta X = X_{\mathrm{excited}} - X_{\mathrm{ground}}$. The Tables in this Section have some values in boldface which correspond the CIPSI wave functions that are used as VMC and DMC trial wave functions either in the SI or in the main text.

## 3.1 Nitroxyl

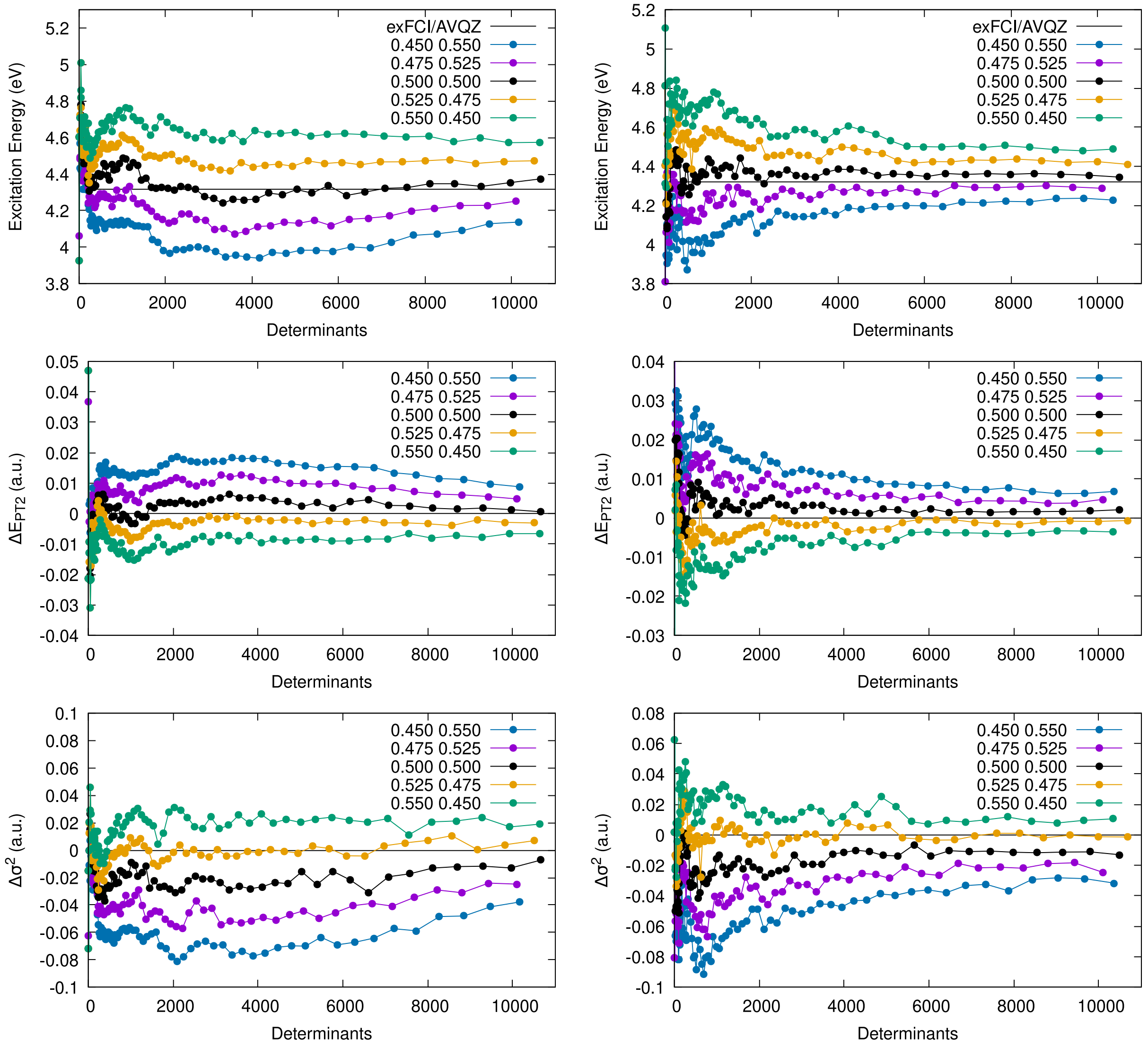


Figure 2: Nitroxyl CIPSI energies, PT2 energies, and variances in the CAS (left) and NO (right) basis, using different input weights ($w_1^{\text{inp}}$ $w_2^{\text{inp}}$).

Table 6: Nitroxyl CIPSI energies ($E_{\mathrm{CIPSI}}$), PT2 energies ($E_{\mathrm{PT2}}$), and variances ($\sigma^2$) of ground- and excited-state CIPSI expansions for different input weights, $w_I^{\mathrm{inp}}$. The CIPSI excitation energy, $\Delta E$, is given in eV, the remaining values are in a.u. The optimal weights ($w_1^{\mathrm{inp}} - w_2^{\mathrm{inp}}$) for both CAS and NO orbitals are 0.525−0.475.

| | $E_{\mathrm{CIPSI}}$ | | | $E_{\mathrm{PT2}}$ | | | $\sigma^2$ | | |
|---|---|---|---|---|---|---|---|---|---|
| # det. | $1^1\mathrm{A}'$ | $2^1\mathrm{A}'$ | $\Delta E$ | $1^1\mathrm{A}'$ | $2^1\mathrm{A}'$ | $\Delta E_{\mathrm{PT2}}$ | $1^1\mathrm{A}'$ | $2^1\mathrm{A}'$ | $\Delta\sigma^2$ |
| | | | | CAS 0.50−0.50 | | | | | |
| 2 | -25.9894 | -25.8203 | 4.602 | -0.5649 | -0.5861 | -0.0212 | 1.5944 | 1.5793 | -0.0151 |
| 59 | -26.0943 | -25.9193 | 4.762 | -0.3468 | -0.3638 | -0.0170 | 1.2972 | 1.3152 | 0.0180 |
| 129 | -26.1106 | -25.9417 | 4.596 | -0.3219 | -0.3300 | -0.0081 | 1.2517 | 1.2527 | 0.0010 |
| **363** | -26.1292 | -25.9708 | **4.310** | -0.2968 | -0.2901 | **0.0067** | 1.1848 | 1.1484 | **-0.0364** |
| 600 | -26.1476 | -25.9841 | 4.448 | -0.2733 | -0.2742 | -0.0009 | 1.1203 | 1.1019 | -0.0184 |
| 1268 | -26.1756 | -26.0124 | 4.441 | -0.2394 | -0.2401 | -0.0007 | 1.0083 | 0.9952 | -0.0131 |
| **1627** | -26.1849 | -26.0241 | **4.374** | -0.2279 | -0.2258 | **0.0021** | 0.9723 | 0.9484 | **-0.0239** |
| 2686 | -26.2087 | -26.0498 | 4.324 | -0.2006 | -0.1971 | 0.0036 | 0.8746 | 0.8542 | -0.0204 |
| **3447** | -26.2206 | -26.0643 | **4.252** | -0.1867 | -0.1813 | **0.0053** | 0.8236 | 0.7975 | **-0.0262** |
| **10660** | -26.2846 | -26.1238 | **4.374** | -0.1205 | -0.1198 | **0.0006** | 0.5826 | 0.5754 | **-0.0072** |
| | | | | CAS 0.525−0.475 | | | | | |
| 2 | -25.9894 | -25.8203 | 4.602 | -0.5649 | -0.5861 | -0.0212 | 1.5944 | 1.5793 | -0.0151 |
| 59 | -26.0943 | -25.9193 | 4.762 | -0.3468 | -0.3638 | -0.0170 | 1.2972 | 1.3152 | 0.0180 |
| 136 | -26.1129 | -25.9412 | 4.672 | -0.3181 | -0.3305 | -0.0124 | 1.2464 | 1.2543 | 0.0079 |
| **322** | -26.1278 | -25.9652 | **4.424** | -0.2986 | -0.2971 | **0.0015** | 1.1891 | 1.1678 | **-0.0213** |
| 600 | -26.1494 | -25.9823 | 4.547 | -0.2710 | -0.2762 | -0.0053 | 1.1122 | 1.1071 | -0.0050 |
| 1235 | -26.1773 | -26.0086 | 4.590 | -0.2370 | -0.2444 | -0.0074 | 0.9993 | 1.0080 | 0.0086 |
| **1569** | -26.1853 | -26.0204 | **4.487** | -0.2273 | -0.2302 | **-0.0029** | 0.9701 | 0.9576 | **-0.0126** |
| 2657 | -26.2108 | -26.0469 | 4.460 | -0.1982 | -0.2002 | -0.0020 | 0.8662 | 0.8628 | -0.0035 |
| **3751** | -26.2287 | -26.0647 | **4.464** | -0.1786 | -0.1809 | **-0.0022** | 0.7961 | 0.7951 | **-0.0010** |
| **10516** | -26.2856 | -26.1213 | **4.473** | -0.1196 | -0.1227 | **-0.0031** | 0.5788 | 0.5858 | **0.0070** |
| | | | | NO 0.525−0.475 | | | | | |
| 2 | -25.9964 | -25.8197 | 4.810 | -0.5825 | -0.6169 | -0.0344 | 1.5820 | 1.5837 | 0.0016 |
| 64 | -26.1015 | -25.9388 | 4.426 | -0.3711 | -0.3672 | 0.0040 | 1.2827 | 1.2704 | -0.0123 |
| 133 | -26.1257 | -25.9575 | 4.576 | -0.3328 | -0.3397 | -0.0068 | 1.1964 | 1.2034 | 0.0070 |
| **321** | -26.1578 | -25.9913 | **4.529** | -0.2879 | -0.2940 | **-0.0062** | 1.0784 | 1.0786 | **0.0002** |
| 617 | -26.1846 | -26.0235 | 4.386 | -0.2522 | -0.2489 | 0.0033 | 0.9877 | 0.9603 | -0.0274 |
| 1257 | -26.2283 | -26.0597 | 4.588 | -0.1966 | -0.2027 | -0.0061 | 0.8156 | 0.8196 | 0.0040 |
| **1573** | -26.2411 | -26.0742 | **4.542** | -0.1812 | -0.1855 | **-0.0042** | 0.7655 | 0.7683 | **0.0027** |
| 2699 | -26.2704 | -26.1066 | 4.455 | -0.1470 | -0.1484 | -0.0014 | 0.6549 | 0.6524 | -0.0026 |
| **2900** | -26.2743 | -26.1102 | **4.466** | -0.1425 | -0.1442 | **-0.0017** | 0.6395 | 0.6379 | **-0.0016** |
| **10690** | -26.3292 | -26.1671 | **4.410** | -0.0813 | -0.0820 | **-0.0006** | 0.4124 | 0.4111 | **-0.0013** |

## 3.2 Glyoxal

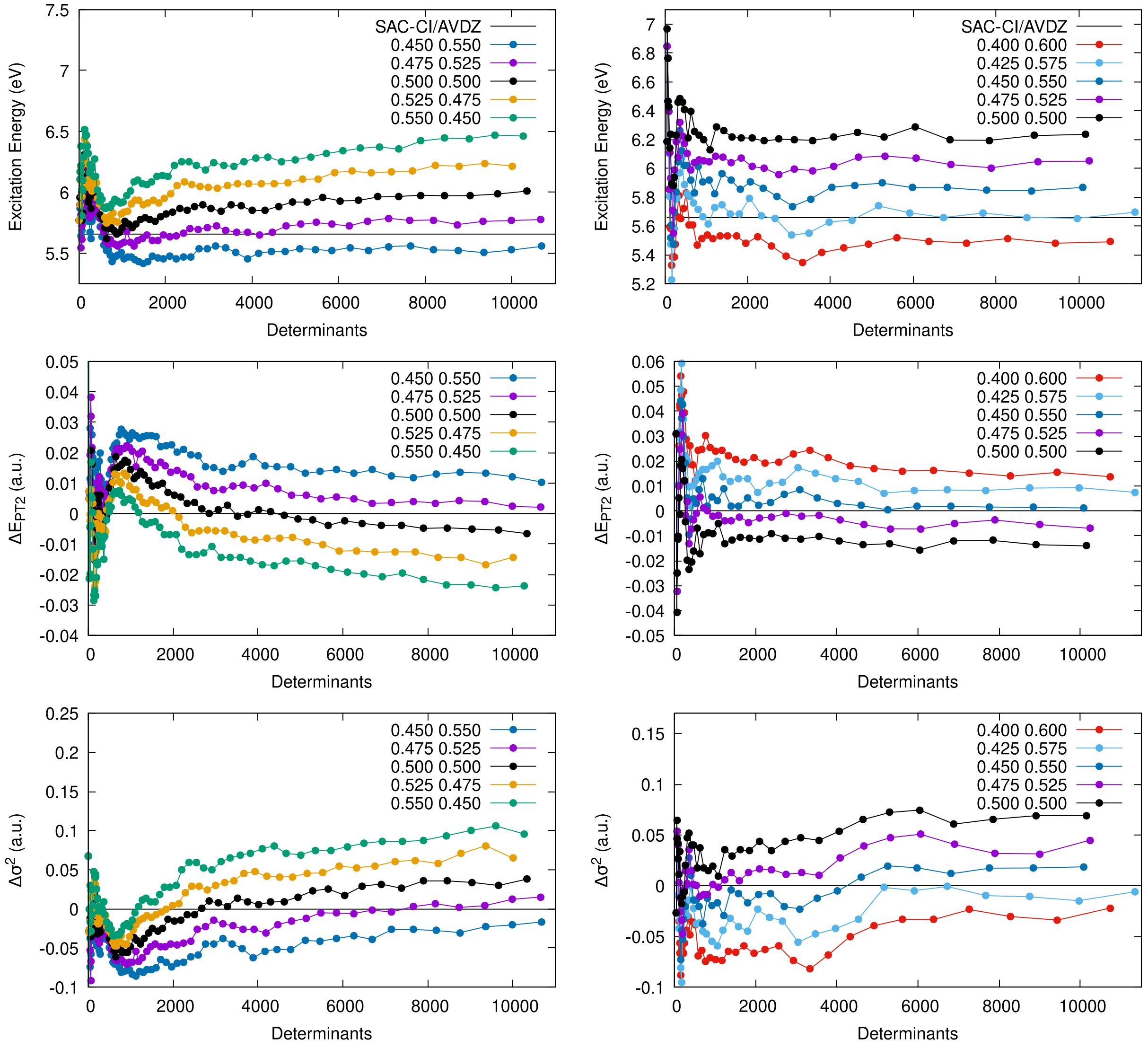


Figure 3: Glyoxal CIPSI energies, PT2 energies, and variances in the CAS (left) and NO (right) basis, using different input weights ($w_1^{\mathrm{inp}}$ $w_2^{\mathrm{inp}}$).

Table 7: Glyoxal CIPSI energies ($E_{\text{CIPSI}}$), PT2 energies ($E_{\text{PT2}}$), and variances ($\sigma^2$) of ground- and excited-state CIPSI expansions using different input weights, $w_I^{\text{inp}}$. The CIPSI excitation energy, $\Delta E$, is given in eV, the remaining values are in a.u. The optimal weights ($w_1^{\text{inp}} - w_2^{\text{inp}}$) for CAS are 0.50−0.50 and for NO are 0.45−0.55.

| | $E_{\text{CIPSI}}$ | | | $E_{\text{PT2}}$ | | | $\sigma^2$ | | |
|---|---|---|---|---|---|---|---|---|---|
| # det. | $1^1A_g$ | $2^1A_g$ | $\Delta E$ | $1^1A_g$ | $2^1A_g$ | $\Delta E_{\text{PT2}}$ | $1^1A_g$ | $2^1A_g$ | $\Delta\sigma^2$ |
| | | | | CAS 0.475−0.525 | | | | | |
| 2 | -43.7144 | -43.4030 | 8.473 | -0.9159 | -1.2042 | -0.2883 | 2.8205 | 2.8883 | 0.0678 |
| 57 | -43.8122 | -43.6084 | 5.543 | -0.6909 | -0.6526 | 0.0383 | 2.5581 | 2.4665 | -0.0916 |
| 127 | -43.8488 | -43.6247 | 6.097 | -0.6275 | -0.6285 | -0.0010 | 2.4248 | 2.4309 | 0.0061 |
| 372 | -43.8848 | -43.6707 | 5.827 | -0.5696 | -0.5626 | 0.0070 | 2.3218 | 2.2899 | -0.0319 |
| 582 | -43.8995 | -43.6910 | 5.675 | -0.5494 | -0.5342 | 0.0151 | 2.2827 | 2.2291 | -0.0536 |
| 1281 | -43.9266 | -43.7224 | 5.557 | -0.5152 | -0.4947 | 0.0205 | 2.1864 | 2.1236 | -0.0628 |
| 1582 | -43.9369 | -43.7293 | 5.649 | -0.5025 | -0.4873 | 0.0152 | 2.1458 | 2.1012 | -0.0446 |
| 2588 | -43.9607 | -43.7510 | 5.706 | -0.4730 | -0.4642 | 0.0088 | 2.0476 | 2.0210 | -0.0266 |
| 3401 | -43.9746 | -43.7655 | 5.689 | -0.4562 | -0.4472 | 0.0090 | 1.9826 | 1.9609 | -0.0217 |
| **10660** | -44.0434 | -43.8311 | **5.777** | -0.3792 | -0.3771 | **0.0021** | 1.7025 | 1.7178 | **0.0153** |
| | | | | CAS 0.50−0.50 | | | | | |
| 2 | -43.7144 | -43.4030 | 8.473 | -0.9159 | -1.2042 | -0.2882 | 2.8205 | 2.8883 | 0.0678 |
| 54 | -43.8122 | -43.6033 | 5.683 | -0.6909 | -0.6616 | 0.0294 | 2.5582 | 2.4928 | -0.0654 |
| 123 | -43.8500 | -43.6220 | 6.204 | -0.6247 | -0.6328 | -0.0081 | 2.4252 | 2.4405 | 0.0153 |
| **358** | -43.8891 | -43.6649 | **6.101** | -0.5636 | -0.5702 | **-0.0065** | 2.3101 | 2.3044 | **-0.0057** |
| 596 | -43.9016 | -43.6907 | 5.740 | -0.5474 | -0.5351 | 0.0122 | 2.2791 | 2.2337 | -0.0454 |
| 1270 | -43.9300 | -43.7186 | 5.752 | -0.5116 | -0.4992 | 0.0124 | 2.1758 | 2.1341 | -0.0416 |
| **1632** | -43.9404 | -43.7285 | **5.766** | -0.4980 | -0.4878 | **0.0102** | 2.1275 | 2.1052 | **-0.0223** |
| 2687 | -43.9673 | -43.7496 | 5.923 | -0.4649 | -0.4649 | -0.0000 | 2.0154 | 2.0196 | 0.0042 |
| **3446** | -43.9792 | -43.7626 | **5.894** | -0.4509 | -0.4511 | **-0.0002** | 1.9632 | 1.9766 | **0.0134** |
| **10342** | -44.0459 | -43.8251 | **6.010** | -0.3768 | -0.3834 | **-0.0066** | 1.6973 | 1.7355 | **0.0382** |
| | | | | NO 0.45−0.55 | | | | | |
| 51 | -43.7834 | -43.5561 | 6.184 | -0.8056 | -0.7745 | 0.0311 | 2.6509 | 2.6242 | -0.0267 |
| 128 | -43.8355 | -43.6183 | 5.911 | -0.6804 | -0.6616 | 0.0188 | 2.4840 | 2.4651 | -0.0189 |
| 349 | -43.8907 | -43.6684 | 6.048 | -0.5890 | -0.5845 | 0.0046 | 2.3171 | 2.3142 | -0.0029 |
| 595 | -43.9187 | -43.6980 | 6.005 | -0.5457 | -0.5416 | 0.0041 | 2.2218 | 2.2100 | -0.0119 |
| 1276 | -43.9594 | -43.7432 | 5.884 | -0.4892 | -0.4831 | 0.0061 | 2.0775 | 2.0565 | -0.0210 |
| **3749** | -44.0262 | -43.8141 | **5.772** | -0.4077 | -0.4010 | **0.0067** | 1.8119 | 1.7876 | **-0.0243** |
| **5046** | -44.0485 | -43.8324 | **5.879** | -0.3823 | -0.3812 | **0.0011** | 1.7075 | 1.7240 | **0.0164** |
| **7040** | -44.0701 | -43.8549 | **5.856** | -0.3586 | -0.3567 | **0.0020** | 1.6296 | 1.6400 | **0.0104** |
| **8642** | -44.0842 | -43.8682 | **5.879** | -0.3446 | -0.3451 | **-0.0005** | 1.5836 | 1.6106 | **0.0270** |
| **10010** | -44.0923 | -43.8785 | **5.817** | -0.3357 | -0.3338 | **0.0019** | 1.5494 | 1.5652 | **0.0158** |
| **12132** | -44.1065 | -43.8913 | **5.858** | -0.3208 | -0.3201 | **0.0007** | 1.5020 | 1.5155 | **0.0134** |
| **15349** | -44.1209 | -43.9080 | **5.793** | -0.3061 | -0.3046 | **0.0015** | 1.4541 | 1.4680 | **0.0139** |

## 3.3 Tetrazine

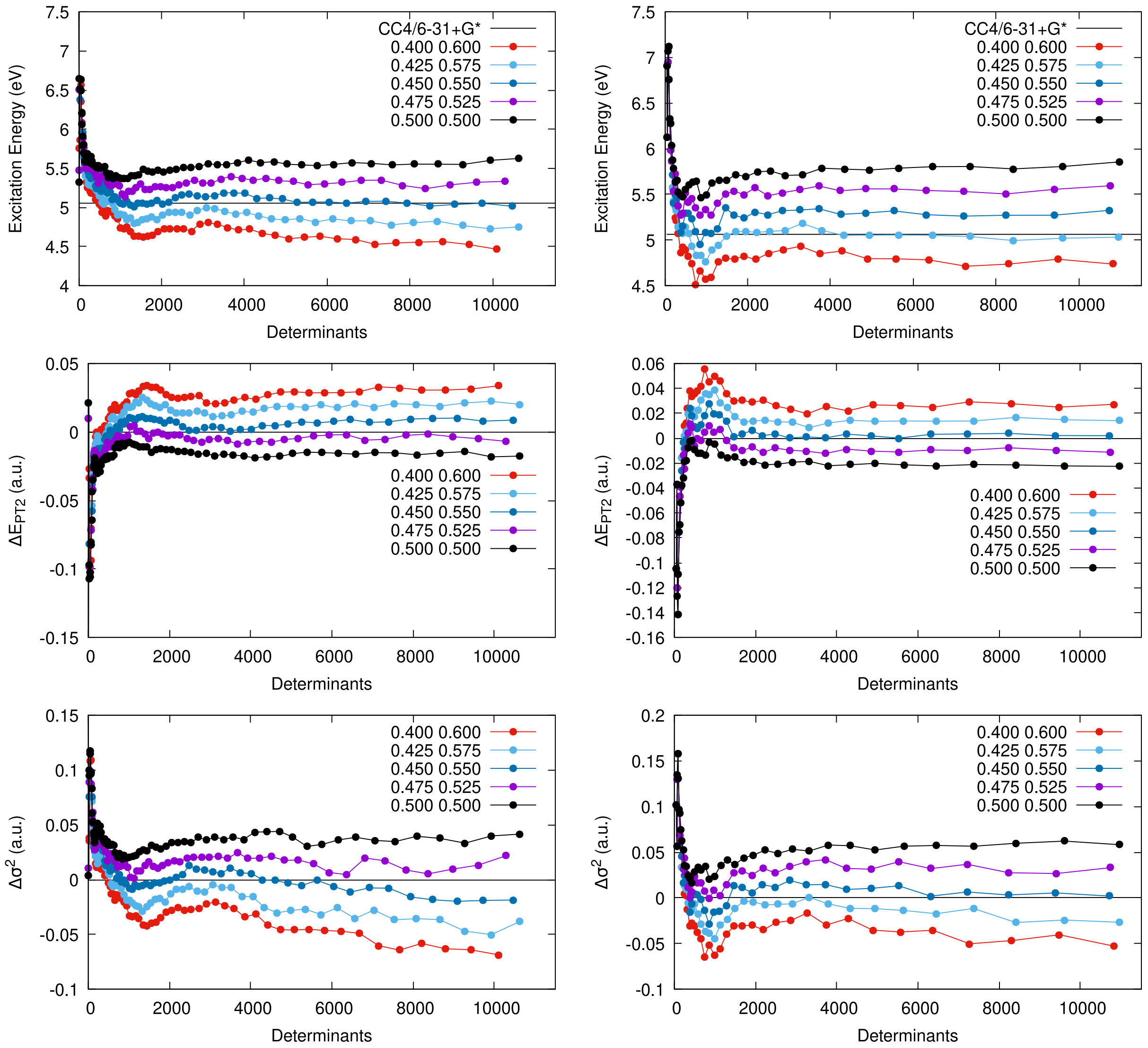


Figure 4: Tetrazine CIPSI energies, PT2 energies, and variances in the CAS (left) and NO (right) basis, using different input weights ($w_1^{\rm inp}$ $w_2^{\rm inp}$).

Table 8: Tetrazine CIPSI energies ($E_{\text{CIPSI}}$), PT2 energies ($E_{\text{PT2}}$), and variances ($\sigma^2$) of ground- and excited-state CIPSI expansions using different input weights, $w_I^{\text{inp}}$. The CIPSI excitation energy, $\Delta E$, is given in eV, the remaining values are in a.u. The optimal weights ($w_1^{\text{inp}} - w_2^{\text{inp}}$) for both CAS and NO are 0.45−0.55.

| | $E_{\text{CIPSI}}$ | | | $E_{\text{PT2}}$ | | | $\sigma^2$ | | |
|---|---|---|---|---|---|---|---|---|---|
| # det. | $1^1\text{A}_{1\text{g}}$ | $2^1\text{A}_{1\text{g}}$ | $\Delta E$ | $1^1\text{A}_{1\text{g}}$ | $2^1\text{A}_{1\text{g}}$ | $\Delta E_{\text{PT2}}$ | $1^1\text{A}_{1\text{g}}$ | $2^1\text{A}_{1\text{g}}$ | $\Delta\sigma^2$ |
| | | | | CAS 0.45−0.55 | | | | | |
| 2 | -51.0258 | -50.7256 | 8.168 | -1.2075 | -1.5672 | -0.3597 | 3.4424 | 3.6206 | 0.1783 |
| 61 | -51.1111 | -50.8776 | 6.354 | -0.9847 | -1.0786 | -0.0939 | 3.2521 | 3.3614 | 0.1093 |
| 136 | -51.1212 | -50.9138 | 5.641 | -0.9667 | -0.9887 | -0.0221 | 3.2319 | 3.2689 | 0.0370 |
| **385** | -51.1388 | -50.9432 | **5.321** | -0.9333 | -0.9413 | **-0.0080** | 3.1883 | 3.2086 | **0.0204** |
| 644 | -51.1510 | -50.9605 | 5.184 | -0.9147 | -0.9125 | 0.0022 | 3.1650 | 3.1700 | 0.0050 |
| **1757** | -51.1878 | -51.0023 | **5.049** | -0.8563 | -0.8473 | **0.0090** | 3.0774 | 3.0759 | **-0.0015** |
| 2895 | -51.2159 | -51.0267 | 5.149 | -0.8156 | -0.8137 | 0.0019 | 3.0145 | 3.0256 | 0.0111 |
| **3718** | -51.2306 | -51.0411 | **5.155** | -0.7962 | -0.7934 | **0.0029** | 2.9851 | 2.9903 | **0.0052** |
| **10470** | -51.2976 | -51.1132 | **5.016** | -0.7087 | -0.7000 | **0.0087** | 2.8173 | 2.7988 | **-0.0185** |
| | | | | NO 0.45−0.55 | | | | | |
| 51 | -51.0780 | -50.8242 | 6.908 | -1.1764 | -1.2809 | -0.1045 | 3.3410 | 3.4428 | 0.1018 |
| 137 | -51.1293 | -50.9092 | 5.989 | -1.0439 | -1.0908 | -0.0468 | 3.2207 | 3.2893 | 0.0686 |
| 377 | -51.1588 | -50.9691 | 5.160 | -0.9881 | -0.9696 | 0.0185 | 3.1552 | 3.1447 | -0.0105 |
| 649 | -51.1860 | -50.9937 | 5.231 | -0.9383 | -0.9278 | 0.0106 | 3.0893 | 3.0874 | -0.0018 |
| 1674 | -51.2464 | –51.051 | 5.305 | -0.8418 | -0.8401 | 0.0017 | 2.9435 | 2.9563 | 0.0128 |
| 2854 | -51.2847 | -51.0898 | 5.303 | -0.7859 | -0.7837 | 0.0022 | 2.8513 | 2.8660 | 0.0146 |
| **3572** | -51.3021 | -51.1065 | **5.324** | -0.7630 | -0.7608 | **0.0022** | 2.8161 | 2.8250 | **0.0088** |
| **7025** | -51.3531 | -51.1590 | **5.282** | -0.6953 | -0.6920 | **0.0033** | 2.6850 | 2.6905 | **0.0055** |
| **10723** | -51.3895 | -51.1937 | **5.326** | -0.6501 | -0.6480 | **0.0021** | 2.5906 | 2.5927 | **0.0020** |

### 3.4 Cyclopentadienone

Data on CAS and NO CIPSI expansions are provided in Table 9 and Figures 5 and 6. Since there are three states to match, we try to achieve a good matching by choosing the input weights manually like for the previous molecules as well as by employing a PT2- and variance-matching algorithm provided in QP (see matching algorithm (ii) in Section 2.1). Using a CAS basis, this algorithm is able to match the PT2 energies of all three states well, but struggles to match the variances of the states simultaneously. Therefore, for the CAS basis, we use input weights to obtain balanced CIPSI expansions. The matching algorithm is instead used for the NO basis since it can simultaneously match all three PT2 energies and variances throughout the expansion.

Table 9: Cyclopentadienone CIPSI energies ($E^{\mathrm{CIPSI}}$), PT2 energies ($E^{\mathrm{PT2}}$), and variances ($\sigma^2$) of ground- and excited-state CIPSI expansions using the matching approach (i) with input weights, $w_I^{\mathrm{inp}}$, and the approach (ii) based on a matching algorithm, with a selection factor of $f = 0.02$. The CIPSI excitation energies, $\Delta E$, are given in eV, the remaining values are in a.u. Only the ground-state energies and variances are listed; the values for the other states can be determined from the differences provided, e.g. $\Delta E_{12} = E(2^1\mathrm{A}_1) - E(1^1\mathrm{A}_1)$.

| | $E^{\mathrm{CIPSI}}$ | | | $E_{\mathrm{PT2}}$ | | | $\sigma^2$ | | |
|---|---|---|---|---|---|---|---|---|---|
| # det. | $1^1\mathrm{A}_1$ | $\Delta E_{12}$ | $\Delta E_{13}$ | $1^1\mathrm{A}_1$ | $\Delta E_{12}^{\mathrm{PT2}}$ | $\Delta E_{13}^{\mathrm{PT2}}$ | $1^1\mathrm{A}_1$ | $\Delta\sigma_{12}^2$ | $\Delta\sigma_{13}^2$ |
| CAS 0.27−0.35−0.38 | | | | | | | | | |
| 98 | -45.7035 | 6.742 | 8.375 | -0.9811 | -0.0341 | -0.0577 | 2.9869 | 0.0513 | 0.0299 |
| 338 | -45.7525 | 6.294 | 8.067 | -0.8629 | 0.0080 | -0.0426 | 2.8731 | 0.0174 | 0.0357 |
| 627 | -45.7566 | 6.237 | 7.827 | -0.8555 | 0.0103 | -0.0343 | 2.8611 | 0.0155 | 0.0252 |
| 1904 | -45.7690 | 6.049 | 7.358 | -0.8354 | 0.0148 | -0.0209 | 2.8172 | 0.0052 | 0.0058 |
| 2507 | -45.7741 | 6.006 | 7.257 | -0.8277 | 0.0159 | -0.0179 | 2.8031 | 0.0050 | 0.0054 |
| **3227** | -45.7808 | **6.011** | **7.169** | -0.8183 | **0.0137** | **-0.0141** | 2.7807 | **0.0118** | **0.0065** |
| **7278** | -45.8074 | **6.006** | **6.981** | -0.7794 | **0.0097** | **-0.0041** | 2.7007 | **0.0183** | **0.0003** |
| **9619** | -45.8176 | **5.997** | **6.909** | -0.7646 | **0.0083** | **-0.0020** | 2.6727 | **0.0199** | **0.0004** |
| CAS | | | | | | | | | |
| 98 | -45.7035 | 6.742 | 8.375 | -0.9811 | -0.0341 | -0.0577 | 2.9869 | 0.0513 | 0.0299 |
| 381 | -45.7534 | 6.343 | 8.008 | -0.8616 | 0.0053 | -0.0410 | 2.8710 | 0.0211 | 0.0343 |
| 670 | -45.7570 | 6.314 | 7.756 | -0.8549 | 0.0058 | -0.0313 | 2.8597 | 0.0225 | 0.0214 |
| **1019** | -45.7597 | **6.273** | **7.499** | -0.8503 | **0.0056** | **-0.0198** | 2.8514 | **0.0192** | **-0.0018** |
| 1729 | -45.7643 | 6.206 | 7.239 | -0.8432 | 0.0068 | -0.0123 | 2.8393 | 0.0145 | -0.0089 |
| 2866 | -45.7687 | 5.979 | 6.873 | -0.8363 | 0.0152 | 0.0020 | 2.8298 | -0.0087 | -0.0355 |
| **3036** | -45.7688 | **5.942** | **6.816** | -0.8362 | **0.0161** | **0.0041** | 2.8294 | **-0.0083** | **-0.0363** |
| 3551 | -45.7711 | 5.880 | 6.705 | -0.8324 | 0.0185 | 0.0094 | 2.8218 | -0.0147 | -0.0447 |
| 5009 | -45.7811 | 5.844 | 6.612 | -0.8180 | 0.0180 | 0.0146 | 2.7895 | -0.0164 | -0.0484 |
| **7248** | -45.7980 | **5.929** | **6.644** | -0.7926 | **0.0116** | **0.0119** | 2.7319 | **0.0029** | **-0.0321** |
| **10198** | -45.8107 | **5.896** | **6.570** | -0.7745 | **0.0126** | **0.0184** | 2.6909 | **0.0028** | **-0.0434** |
| NO | | | | | | | | | |
| 102 | -45.7117 | 6.656 | 8.593 | -1.0544 | -0.0388 | -0.1020 | 2.9870 | 0.0513 | 0.0299 |
| 378 | -45.7654 | 6.329 | 7.500 | -0.9294 | -0.0110 | -0.0317 | 2.8498 | 0.0347 | 0.0325 |
| 670 | -45.7710 | 5.988 | 7.025 | -0.9183 | 0.0095 | -0.0026 | 2.8338 | 0.0060 | -0.0050 |
| **1025** | -45.7815 | **5.950** | **6.854** | -0.8984 | **0.0096** | **0.0069** | 2.807 | **-0.0006** | **-0.0270** |
| 1728 | -45.8040 | 5.979 | 6.836 | -0.8600 | 0.0072 | 0.0079 | 2.7480 | -0.0011 | -0.0307 |
| 2834 | -45.8311 | 6.091 | 6.947 | -0.8153 | 0.0010 | 0.0009 | 2.6698 | -0.0021 | -0.0260 |
| **3033** | -45.8347 | **6.141** | **6.967** | -0.8102 | **-0.0019** | **-0.0001** | 2.6524 | **0.0135** | **-0.0137** |
| 3488 | -45.8416 | 6.124 | 6.972 | -0.7992 | -0.0001 | 0.0006 | 2.6316 | 0.0087 | -0.0156 |
| 4941 | -45.8589 | 6.095 | 6.948 | -0.7727 | 0.0024 | 0.0003 | 2.5790 | 0.0016 | -0.0119 |
| **7151** | -45.8790 | **6.090** | **6.942** | -0.7415 | **0.0006** | **0.0002** | 2.5116 | **0.0156** | **-0.0079** |
| **10206** | -45.8989 | **6.050** | **6.917** | -0.7133 | **0.0038** | **0.0014** | 2.4586 | **0.0071** | **-0.0092** |

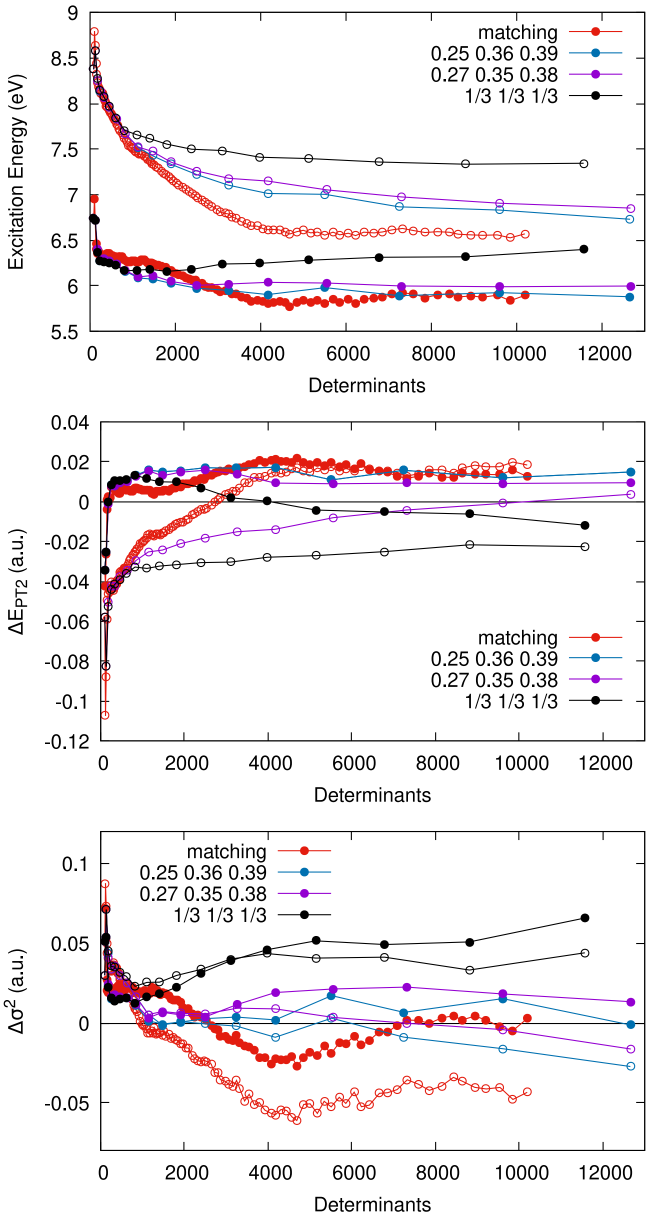


Figure 5: Cyclopentadienone CIPSI energies, PT2 energies, and variances in the CAS basis, obtained using different input weights ($w_1^{\text{inp}}$ $w_2^{\text{inp}}$ $w_3^{\text{inp}}$) as well as the PT2- and variance-matching algorithm (ii) (labeled 'matching'). Filled points represent $\Delta X_{12}$ values while empty points represent $\Delta X_{13}$ values.

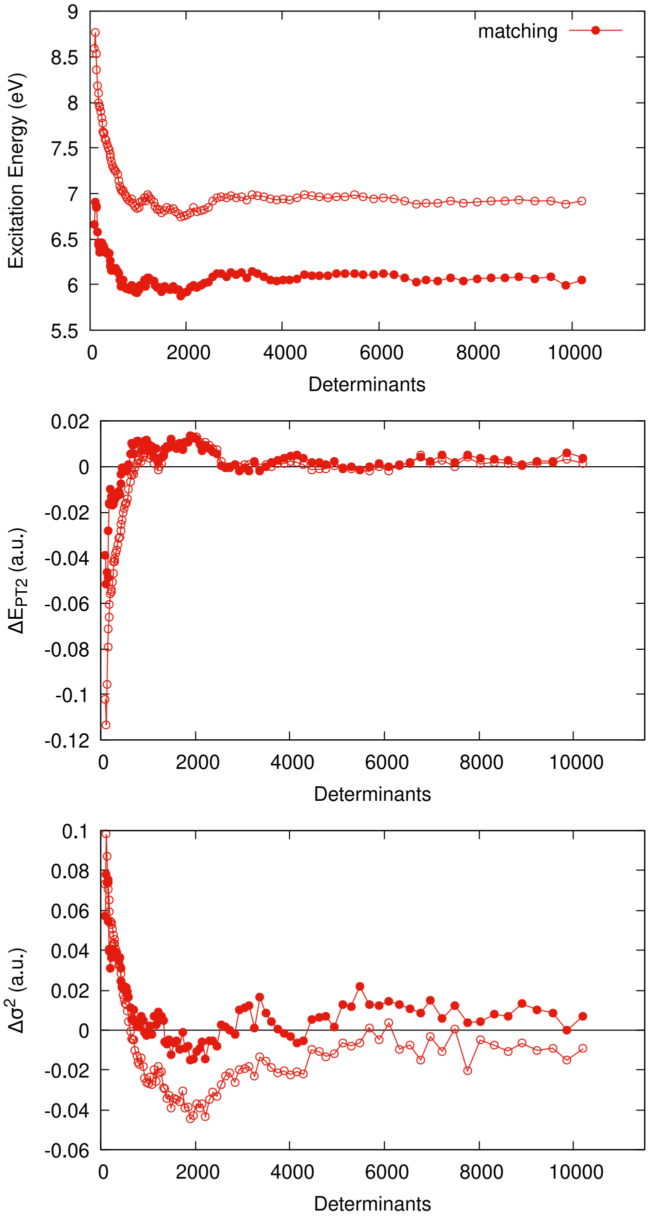


Figure 6: Cyclopentadienone CIPSI energies, PT2 energies, and variances in the NO basis, obtained using the PT2- and variance-matching algorithm (ii) (labeled 'matching'). Filled points represent $\Delta X_{12}$ values while empty points represent $\Delta X_{13}$ values.

# 4 More QMC Data

Tables 10−13 show VMC and DMC total energies (a.u.) and excitation energies (eV). The number of determinants (# det) and parameters (# parm) in each trial wave function is also given. All calculations use BFD pseudopotentials with the corresponding aug-cc-pVDZ basis set.

## 4.1 Nitroxyl

Table 10: Nitroxyl VMC and DMC results. The optimal weights ($w_1^{\rm inp} - w_2^{\rm inp}$) in the CAS basis are 0.525−0.475. Results for weights of 0.50−0.50 are also provided. The optimal weights in the NO basis are 0.50−0.50.

| | | | VMC | | | DMC | | |
|---|---|---|---|---|---|---|---|---|
| WF | # det | # parm | $E$ ($1^1$A′) | $E$ ($2^1$A′) | $\Delta E$ | $E$ ($1^1$A′) | $E$ ($2^1$A′) | $\Delta E$ |
| CAS(2,2) | 2 | 231 | -26.4462(3) | -26.2764(3) | 4.61(1) | -26.4990(2) | -26.3321(2) | 4.54(1) |
| CAS(12,9) | 3528 | 1598 | -26.4762(2) | -26.3134(2) | 4.43(1) | -26.5114(2) | -26.3510(2) | 4.36(1) |
| CIPSI (CAS) | | | | | | | | |
| 0.50−0.50 | 363 | 965 | -26.4876(2) | -26.3236(2) | 4.46(1) | -26.5161(2) | -26.3547(2) | 4.39(1) |
| | 1627 | 2150 | -26.5013(2) | -26.3426(2) | 4.32(1) | -26.5228(2) | -26.3643(2) | 4.31(1) |
| | 3447 | 2986 | -26.5061(2) | -26.3469(2) | 4.33(1) | -26.5248(2) | -26.3661(2) | 4.32(1) |
| | 10660 | 5826 | -26.5106(2) | -26.3506(2) | 4.35(1) | -26.5267(2) | -26.3675(2) | 4.33(1) |
| 0.525−0.475 | 322 | 909 | -26.4891(2) | -26.3271(2) | 4.41(1) | -26.5155(2) | -26.3565(2) | 4.33(1) |
| | 1569 | 2126 | -26.5021(2) | -26.3423(2) | 4.35(1) | -26.5228(2) | -26.3644(2) | 4.31(1) |
| | 3751 | 3135 | -26.5088(2) | -26.3481(2) | 4.37(1) | -26.5260(2) | -26.3667(2) | 4.33(1) |
| | 7464 | 4618 | -26.5111(2) | -26.3511(2) | 4.35(1) | -26.5273(2) | -26.3682(2) | 4.33(1) |
| | 10516 | 5772 | -26.5119(2) | -26.3517(2) | 4.36(1) | -26.5275(2) | -26.3680(2) | 4.34(1) |
| CIPSI (NO) | | | | | | | | |
| 0.50−0.50 | 321 | 850 | -26.4917(2) | -26.3322(2) | 4.34(1) | -26.5164(2) | -26.3583(2) | 4.30(1) |
| | 1573 | 1446 | -26.5001(2) | -26.3415(2) | 4.31(1) | -26.5220(2) | -26.3636(2) | 4.31(1) |
| | 2900 | 2002 | -26.5040(2) | -26.3447(2) | 4.34(1) | -26.5240(2) | -26.3652(2) | 4.32(1) |
| | 7171 | 4124 | -26.5102(2) | -26.3498(2) | 4.36(1) | -26.5267(2) | -26.3677(2) | 4.33(1) |
| | 10690 | 5712 | -26.5113(2) | -26.3519(2) | 4.34(1) | -26.5273(2) | -26.3686(2) | 4.32(1) |
| TBE[a] | | | | | | | | 4.33 |
| CC3/AVQZ[b] | | | | | | | | 5.23 |
| CC4/AVDZ[c] | | | | | | | | 4.454 |
| CC4/AVTZ[c] | | | | | | | | 4.380 |
| CCSDT/AVDZ[b] | | | | | | | | 4.756 |
| CCSDTQ/AVDZ[b] | | | | | | | | 4.424 |
| CCSDTQ/AVTZ[c] | | | | | | | | 4.364 |
| exFCI/AVQZ[d] | | | | | | | | 4.32(0) |
| CASPT2(12,9)/AVQZ[d] | | | | | | | | 4.34 |
| PC-NEVPT2(12,9)/AVQZ[d] | | | | | | | | 4.35 |

[a]Value is ‘safe’, as defined in Ref. 2. FCI/AVTZ
[b]Ref. 7.
[c]Ref. 8.
[d]Ref. 3.

## 4.2 Glyoxal

Table 11: Glyoxal VMC and DMC results. The optimal weights ($w_1^{\mathrm{inp}} - w_2^{\mathrm{inp}}$) in the CAS basis are 0.50−0.50 (< 5,000 determinants) and 0.475−0.525 (> 5,000 determinants). The optimal weights in the NO basis are 0.45−0.55.

| | | | VMC | | | DMC | | |
|---|---|---|---|---|---|---|---|---|
| WF | # det | # parm | $E$ ($1^1\mathrm{A_g}$) | $E$ ($2^1\mathrm{A_g}$) | $\Delta E$ | $E$ ($1^1\mathrm{A_g}$) | $E$ ($2^1\mathrm{A_g}$) | $\Delta E$ |
| CAS(8,6) | 61 | 441 | -44.5603(2) | -44.3473(2) | 5.79(1) | -44.6428(3) | -44.4315(3) | 5.75(1) |
| CIPSI (CAS) | | | | | | | | |
| 0.50−0.50 | 358 | 816 | -44.5729(2) | -44.3633(2) | 5.70(1) | -44.6437(2) | -44.4369(2) | 5.63(1) |
| | 1632 | 2683 | -44.5950(2) | -44.3832(2) | 5.76(1) | -44.6514(3) | -44.4434(3) | 5.66(1) |
| | 3446 | 3903 | -44.6075(2) | -44.3965(2) | 5.74(1) | -44.6565(2) | -44.4494(2) | 5.64(1) |
| | 10342 | 7248 | -44.6273(2) | -44.4124(2) | 5.85(1) | -44.6663(2) | -44.4567(2) | 5.70(1) |
| 0.475−0.525 | 10660 | 7374 | -44.6288(2) | -44.4160(2) | 5.79(1) | -44.6675(2) | -44.4585(2) | 5.69(1) |
| CIPSI (NO) | | | | | | | | |
| 0.45−0.55 | 3749 | 2908 | -44.6072(2) | -44.3957(2) | 5.76(1) | -44.6564(2) | -44.4482(2) | 5.67(1) |
| | 5046 | 3498 | -44.6127(2) | -44.4004(2) | 5.78(1) | -44.6600(2) | -44.4509(2) | 5.69(1) |
| | 7040 | 4500 | -44.6150(2) | -44.4048(2) | 5.72(1) | -44.6613(2) | -44.4534(2) | 5.66(1) |
| | 8642 | 5433 | -44.6181(2) | -44.4098(2) | 5.67(1) | -44.6632(2) | -44.4559(2) | 5.64(1) |
| | 10010 | 6188 | -44.6205(2) | -44.4114(2) | 5.69(1) | -44.6635(2) | -44.4567(2) | 5.63(1) |
| | 12132 | 6269 | -44.6237(2) | -44.4145(2) | 5.70(1) | -44.6654(2) | -44.4576(2) | 5.66(1) |
| | 15349 | 8577 | -44.6276(2) | -44.4180(2) | 5.70(1) | -44.6670(2) | -44.4600(2) | 5.63(1) |
| TBE[a] | | | | | | | | 5.61 |
| CC3/AVQZ[b] | | | | | | | | 6.76 |
| CC4/6-31+G*[c] | | | | | | | | 5.699 |
| CC4/AVDZ[c] | | | | | | | | 5.593 |
| CCSDTQ/6-31+G*[c] | | | | | | | | 5.670 |
| exFCI/AVDZ | | | | | | | | 5.56(11) |
| SAC-CI/AVDZ[d] | | | | | | | | 5.66 |
| CASPT2(14,12)/AVQZ[b] | | | | | | | | 5.43 |
| PC-NEVPT2(14,12)/AVQZ[b] | | | | | | | | 5.52 |

[a]This value is ‘safe’, as defined in Ref. 2. exFCI/AVDZ + ( CCSDT/AVTZ - CCSDT/AVDZ )
[b]Ref. 3.
[c]Ref. 8.
[d]Ref. 9. See reference for basis set details.

## 4.3 Tetrazine

Table 12: Tetrazine VMC and DMC results. The optimal weights ($w_1^{\rm inp} - w_2^{\rm inp}$) in the CAS and NO basis are 0.45−0.55.

| WF | # det | # parm | VMC $E$ ($1^1$A$_{1g}$) | VMC $E$ ($2^1$A$_{1g}$) | VMC $\Delta E$ | DMC $E$ ($1^1$A$_{1g}$) | DMC $E$ ($2^1$A$_{1g}$) | DMC $\Delta E$ |
|---|---|---|---|---|---|---|---|---|
| CAS(4,4) | 6 | 399 | -52.1492(2) | -51.9510(2) | 5.39(1) | -52.2647(3) | -52.0734(3) | 5.21(1) |
| CAS(8,6) | 33 | 414 | -52.1649(2) | -51.9569(2) | 5.66(1) | -52.2725(3) | -52.0767(3) | 5.33(1) |
| CAS(12,8) | 112 | 454 | -52.1652(2) | -51.9658(2) | 5.43(1) | -52.2740(3) | -52.0824(3) | 5.21(1) |
| CAS(14,10) | 1824 | 1078 | -52.1772(2) | -51.9880(2) | 5.15(1) | -52.2804(3) | -52.0927(3) | 5.11(1) |
| CIPSI (CAS) | | | | | | | | |
| 0.45−0.55 | 385 | 871 | -52.1826(2) | -52.0008(2) | 4.95(1) | -52.2811(3) | -52.0985(3) | 4.97(1) |
| | 1757 | 1608 | -52.1966(2) | -52.0203(2) | 4.80(1) | -52.2841(2) | -52.1042(2) | 4.89(1) |
| | 3718 | 2302 | -52.2138(2) | -52.0323(2) | 4.94(1) | -52.2918(3) | -52.1089(3) | 4.98(1) |
| | 10470 | 6934 | -52.2321(2) | -52.0504(2) | 4.94(1) | -52.2996(2) | -52.1171(2) | 4.97(1) |
| CIPSI (NO) | | | | | | | | |
| 0.45−0.55 | 3572 | 2414 | -52.2198(2) | -52.0346(2) | 5.04(1) | -52.2935(3) | -52.1098(3) | 5.00(1) |
| | 7025 | 4141 | -52.2300(2) | -52.0451(2) | 5.03(1) | -52.2981(3) | -52.1148(3) | 4.99(1) |
| | 10723 | 6053 | -52.2379(2) | -52.0528(2) | 5.04(1) | -52.3015(3) | -52.1182(3) | 4.99(1) |
| TBE[a] | | | | | | | | 4.61 |
| CC3/6-31+G*[b] | | | | | | | | 6.22 |
| CC3/AVQZ[b] | | | | | | | | 6.19 |
| CC4/6-31+G*[c] | | | | | | | | 5.06 |
| CCSDT/AVTZ[b] | | | | | | | | 5.96 |
| exFCI/AVDZ | | | | | | | | 5.15(3) |
| CASPT2(14,10)/AVQZ[b] | | | | | | | | 4.68 |
| PC-NEVPT2(14,10)/AVQZ[b] | | | | | | | | 4.60 |

[a]This value is ‘unsafe’, as defined in Ref 2. NEVTPT2/AVTZ
[b]Ref. 3.
[c]Ref. 8.

## 4.4 Cyclopentadienone

Table 13: Cyclopentadienone VMC and DMC results. The optimal weights ($w_1^{\text{inp}} - w_2^{\text{inp}} - w_3^{\text{inp}}$) in the CAS basis are 0.27−0.35−0.38. All optimizations use $\lambda_{IJ} = 1.0$ unless specified otherwise.

| | | | VMC | | | | | | DMC | | | | | |
|---|---|---|---|---|---|---|---|---|---|---|---|---|---|---|
| WF | # det | # parm | $E$ ($1^1$A$_1$) | $E$ ($2^1$A$_1$) | $E$ ($3^1$A$_1$) | $\Delta E_{12}$ | $\Delta E_{13}$ | $\Delta E_{23}$ | $E$ ($1^1$A$_1$) | $E$ ($2^1$A$_1$) | $E$ ($3^1$A$_1$) | $\Delta E_{12}$ | $\Delta E_{13}$ | $\Delta E_{23}$ |
| CAS(6,6) | 208 | 967 | -46.7358(1) | -46.5119(1) | -46.4438(1) | 6.093(4) | 7.947(4) | 1.854(4) | -46.8322(1) | -46.6109(1) | -46.5439(1) | 6.021(6) | 7.844(6) | 1.822(6) |
| CIPSI (CAS) | | | | | | | | | | | | | | |
| 0.27−0.35−0.38 | 9619[e] | 10062 | -46.7794(2) | -46.5591(2) | -46.5220(2) | 6.00(1) | 7.00(1) | 1.01(1) | -46.8488(2) | -46.6329(2) | -46.5967(2) | 5.87(1) | 6.86(1) | 0.99(1) |
| matching ($f$: 0.02) | 1019[e] | 4735 | -46.7532(2) | -46.5281(2) | -46.4930(2) | 6.13(1) | 7.08(1) | 0.96(1) | -46.8405(2) | -46.6204(2) | -46.5854(2) | 5.99(1) | 6.94(1) | 0.95(1) |
| | 3036[e] | 6235 | -46.7663(2) | -46.5440(2) | -46.5099(2) | 6.05(1) | 6.98(1) | 0.93(1) | -46.8448(2) | -46.6278(2) | -46.5938(2) | 5.91(1) | 6.83(1) | 0.92(1) |
| | 7248 | 9008 | -46.7741(2) | -46.5537(2) | -46.5200(2) | 6.00(1) | 6.91(1) | 0.92(1) | -46.8472(2) | -46.6310(2) | -46.5972(2) | 5.88(1) | 6.80(1) | 0.92(1) |
| | 10198 | 10557 | -46.7807(2) | -46.5605(2) | -46.5263(2) | 5.99(1) | 6.92(1) | 0.93(1) | -46.8503(2) | -46.6330(2) | -46.5988(2) | 5.90(1) | 6.84(1) | 0.99(1) |
| CIPSI (NO) | | | | | | | | | | | | | | |
| matching ($f$: 0.02) | 1025[e] | 2403 | -46.7542(2) | -46.5361(2) | -46.5000(2) | 5.94(1) | 6.92(1) | 0.98(1) | -46.8389(2) | -46.6222(2) | -46.5864(2) | 5.90(1) | 6.87(1) | 0.97(1) |
| | 3033[e] | 3645 | -46.7670(2) | -46.5485(2) | -46.5112(2) | 5.95(1) | 6.96(1) | 1.01(1) | -46.8432(2) | -46.6276(2) | -46.5908(2) | 5.87(1) | 6.87(1) | 1.00(1) |
| | 7151 | 5365 | -46.7826(2) | -46.5610(2) | -46.5236(2) | 6.03(1) | 7.05(1) | 1.02(1) | -46.8480(2) | -46.6302(2) | -46.5937(2) | 5.92(1) | 6.91(1) | 0.99(1) |
| | 10206 | 6574 | -46.7874(2) | -46.5656(2) | -46.5292(2) | 6.04(1) | 7.03(1) | 0.99(1) | -46.8498(2) | -46.6330(2) | -46.5965(2) | 5.90(1) | 6.89(1) | 0.99(1) |
| TBE[a] | | | | | | | | | | | | 6.00[b] | 6.09[c] | 0.09 |
| ADC(3)/AVTZ | | | | | | | | | | | | 4.59[d] | 6.50[d] | 1.91 |
| CC2/AVTZ | | | | | | | | | | | | | 6.50[d] | |
| CC3/AVTZ | | | | | | | | | | | | 7.10[d] | 6.21[d] | -0.89 |
| CCSD/AVTZ | | | | | | | | | | | | | 6.68[d] | |
| CCSDT-3/AVTZ | | | | | | | | | | | | | 6.33[d] | |
| exFCI/AVDZ | | | | | | | | | | | | 5.81(39) | 6.93(24) | 1.10(13) |

[a]This value is 'unsafe' as defined in Ref. 2.
[b]NEVPT2/AVTZ.
[c]CCSDT/AVDZ + ( CC3/AVTZ-CC3/AVDZ ).
[d]Values are also 'unsafe'.
[e]Optimization used $(\lambda_{12}, \lambda_{13}, \lambda_{23}) = (1.5, 2.0, 1.0)$

# 5 State-specific VMC optimization

## 5.1 Gradient Derivation

The state-specific optimization method used here minimizes the objective function

$$O_I[\Psi_I] = E_I[\Psi_I] + \sum_{J \neq I} \lambda_{IJ} |S_{IJ}|^2, \tag{11}$$

where $E_I$ is given by,

$$E_I = \frac{\langle \Psi_I | H | \Psi_I \rangle}{\langle \Psi_I | \Psi_I \rangle} = \langle E_{\mathrm{L}}^I (\mathbf{R}) \rangle_{\mathbf{R} \sim |\Psi_I(\mathbf{R})|^2}, \tag{12}$$

For a VMC calculation over multiple states, a guiding function is introduced, $\Psi_{\mathrm{g}} = \sqrt{\sum_I \gamma_I |\Psi_I|^2}$ and one computes the energies of the states as

$$E_I = \frac{\left\langle t_I E_{\mathrm{L}}^I \right\rangle_{\mathbf{R} \sim \rho_{\mathrm{g}}(\mathbf{R})}}{\left\langle t_I \right\rangle_{\mathbf{R} \sim \rho_{\mathrm{g}}(\mathbf{R})}}, \tag{13}$$

where $E_{\mathrm{L}}^I$ is the local energy of state $I$ and $t_I = |\Psi_I / \Psi_{\mathrm{g}}|^2$.

Since the relative norm of the different wave functions may change during the optimization, we must ensure that this is taken into account in the definition of the guiding wave function so that all states are sampled with similar efficiency. Therefore, we use as a guiding wave function at optimization step $n+1$

$$|\Psi_{\mathrm{g}}^{n+1}|^2 = |\Psi_1^{n+1}|^2 + \sum_{I=2}^{N_{\mathrm{states}}} \frac{\left\langle \tilde{t}_1^{n+1} \right\rangle_{\mathbf{R} \sim \rho_{\mathrm{g}}^n(\mathbf{R})}}{\left\langle \tilde{t}_I^{n+1} \right\rangle_{\mathbf{R} \sim \rho_{\mathrm{g}}^n(\mathbf{R})}} |\Psi_I^{n+1}|^2, \tag{14}$$

where $\tilde{t}_I^{n+1}$ is an approximation to the norm of $\Psi_I^{n+1}$ using the first-order estimate $\tilde{\Psi}_I^{n+1} = \Psi_I^n + \sum_j^{\mathrm{N_{parm}}} \delta p_{Ij} \partial_{p_{Ij}} \Psi_I^n$ with $\delta p_{Ij}$ a parameter variation, $\partial_{p_{Ij}} \Psi_I^n$ the derivative of the wave function with respect parameter $p_{Ij}$, and $N_{\mathrm{parm}}$ the number of parameters.

Taking the derivative of Eq. 11 with respect to a parameter of state $I$, $p_I$, gives

$$\begin{aligned}\frac{\partial O_I}{\partial p_I} &= 2\left\{\left\langle t_I E_{\mathrm{L}}^I \frac{\partial}{\partial p_I}\ln\Psi_I\right\rangle \langle t_I\rangle^{-1} - \langle t_I E_{\mathrm{L}}^I\rangle \left\langle t_I \frac{\partial}{\partial p_I}\ln\Psi_I\right\rangle \langle t_I\rangle^{-2}\right\} \\ &+ 2\sum_{J\neq I}\lambda_{IJ}\left\{\left\langle \frac{\Psi_I}{\Psi_{\mathrm{g}}}\frac{\Psi_J}{\Psi_{\mathrm{g}}}\frac{\partial}{\partial p_I}\ln\Psi_I\right\rangle \frac{\left\langle \frac{\Psi_I}{\Psi_{\mathrm{g}}}\frac{\Psi_J}{\Psi_{\mathrm{g}}}\right\rangle}{\langle t_I\rangle\langle t_J\rangle} - \left\langle t_I\frac{\partial}{\partial p_I}\ln\Psi_I\right\rangle \frac{\left\langle \frac{\Psi_I}{\Psi_{\mathrm{g}}}\frac{\Psi_J}{\Psi_{\mathrm{g}}}\right\rangle^2}{\langle t_I\rangle\langle t_J\rangle}\langle t_I\rangle^{-1}\right\},\end{aligned} \tag{15}$$

where $\langle\cdot\rangle$ implies the average over configurations sampled from $\rho_{\mathrm{g}}(\mathbf{R})$. Therefore, the gradient has two terms

$$\frac{\partial O_I}{\partial p_I} = [g_{\mathrm{tot}}]_I = [g_{\mathrm{E}} + g_{\mathrm{O}}]_I\,, \tag{16}$$

where the first term, $g_{\mathrm{E}}$, is the derivative of the energy (Eq. 13) while the second term, $g_{\mathrm{O}}$, is the derivative of the orthogonality penalty (the second term in Eq. 11). The term $g_{\mathrm{O}}$ can be rewritten as

$$[g_{\mathrm{O}}]_I = 2\sum_{J\neq I}\lambda_{IJ}|S_{IJ}|^2\left\{\left\langle \frac{\Psi_I}{\Psi_{\mathrm{g}}}\frac{\Psi_J}{\Psi_{\mathrm{g}}}\frac{\partial}{\partial p_I}\ln\Psi_I\right\rangle\left\langle \frac{\Psi_I}{\Psi_{\mathrm{g}}}\frac{\Psi_J}{\Psi_{\mathrm{g}}}\right\rangle^{-1} - \left\langle t_I\frac{\partial}{\partial p_I}\ln\Psi_I\right\rangle\langle t_I\rangle^{-1}\right\}, \tag{17}$$

using that the squared overlap is given by

$$|S_{IJ}|^2 = \frac{\left\langle \frac{\Psi_I}{\Psi_{\mathrm{g}}}\frac{\Psi_J}{\Psi_{\mathrm{g}}}\right\rangle^2}{\left\langle \frac{\Psi_I^2}{\Psi_{\mathrm{g}}^2}\right\rangle\left\langle \frac{\Psi_J^2}{\Psi_{\mathrm{g}}^2}\right\rangle} = \frac{\left\langle \frac{\Psi_I}{\Psi_{\mathrm{g}}}\frac{\Psi_J}{\Psi_{\mathrm{g}}}\right\rangle^2}{\langle t_I\rangle\langle t_J\rangle}. \tag{18}$$

For each state $I$, the total gradient is then used in the stochastic reconfiguration (SR) equations,

$$\bar{S}^{(I)}\Delta p_I = -\tau[g_{\mathrm{tot}}]_I, \tag{19}$$

where $\bar{S}^{(I)}$ is related to the overlap matrix between the derivatives of the wave function of state $I$ with respect to its parameters.

## 5.2 Optimization of 2-determinant wave function of nitroxyl

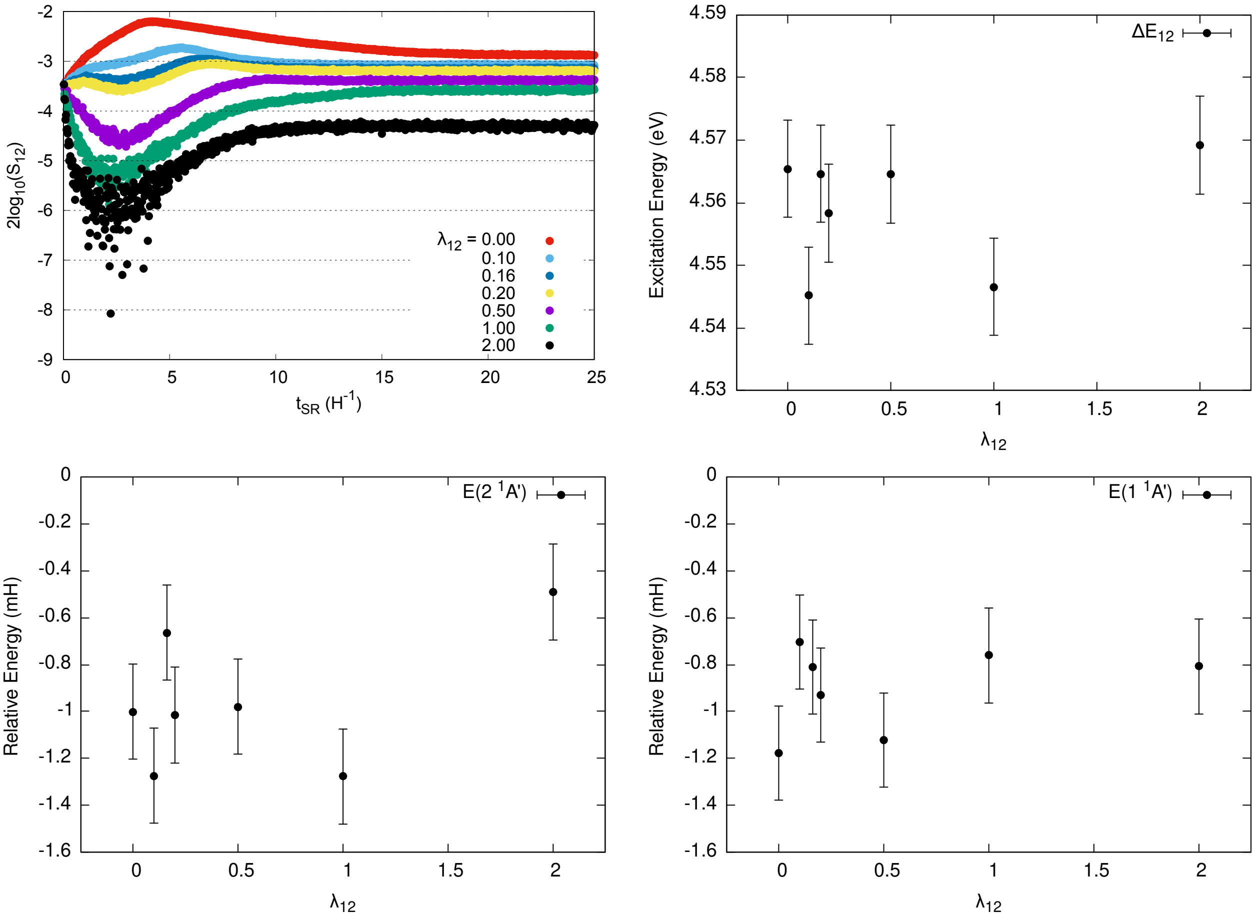


Figure 7: Effect of choice of $\lambda_{12}$ on the VMC optimization of a 2-determinant wave function for the ground and excited states of nitroxyl. The starting wave function comprises the HF and the relevant doubly-excited determinant, expressed on the CAS orbitals. Top-left: The squared overlap of the ground and excited states ($|S_{12}|^2$) plotted along the SR optimization for different values of $\lambda_{12}$ (a SR time-step of 0.025 $H^{-1}$ is used). Top-right: Final VMC excitation energies obtained with different $\lambda_{12}$. Bottom: Final VMC energies of the ground (left) and excited state (right) shifted by 26.4460 H and 26.2784 H, respectively, using different $\lambda_{12}$.

The 2-determinant wave function of nitroxyl studied in Figure 7 shows that, without any penalty on the energy ($\lambda_{12} = 0$), the excited state does not collapse to the ground state. This is due to the orbital symmetries in the dominant determinant of the double excitation. The doubly-excited determinant promotes both electrons to an orbital of different symmetry than the original ($a' \rightarrow a''$) and orbital optimization cannot transform the doubly-excited determinant into the HF one. In this case, $|S_{IJ}|^2$ always plateaus, and increasing $\lambda_{12}$ reduces the value at which it plateaus. All values

of the excitation energies (top-right of Figure 7) are compatible within the error bars.

## 5.3 Analysis of $\lambda_{IJ}$ in a 3-state nitroxyl optimization

We optimize a 3-state nitroxyl system using different sets of $\lambda_{IJ}$. In addition to what is reported in the main text, we consider a third excited state of symmetry $^1\mathrm{A}'$ and analyze how the choice of $\lambda_{IJ}$ impacts the overlaps and the excitation energies of the three states.

In the VMC optimization, the starting determinant components of the wave functions consist of 3040 determinants and are from a CIPSI expansion over 3 states in a SA(3)-CASSCF(12,9) orbital basis. The PT2 energies and variances of the 3 states are matched using the built-in matching algorithm in QP, explained in Section 2.1. The values of $\Delta E_{12}^{\mathrm{CIPSI}}$, $\Delta E_{13}^{\mathrm{CIPSI}}$, $\Delta E_{12}^{\mathrm{PT2}}$, $\Delta E_{13}^{\mathrm{PT2}}$, $\Delta\sigma_{12}^2$, and $\Delta\sigma_{123}^2$ are given by 4.414 eV, 6.137 eV, $-0.0008$ a.u., 0.0018 a.u., $-0.0090$ a.u., and 0.0060 a.u., respectively.

We use similar values of $\lambda_{IJ}$ as in the analysis of the 3-state cyclopentadienone system. As shown in Figure 8 (panel a), the overlaps are overall lower than the values obtained for cyclopentadienone with a similar determinant expansion (shown in the top left corner). The set of $\lambda_{IJ}$ which gives the largest overlaps is $\lambda^{(3)} = (1.0, 1.0, 1.0)$. The overlap $|S_{23}|^2$ remains stable for all choices of $\lambda_{IJ}$. The overlap $|S_{13}|^2$ is consistently the largest but is easier to stabilize than $|S_{12}|^2$, which appears instead to plateau only later in the variational optimization, depending on the choice of $\lambda_{12}$. For one $\lambda_{12}$ ($\lambda_{12}^{(8)} = 1.75$) where a plateau is observed early in the optimization run, we perform more iterations and find that $|S_{12}|^2$ stabilizes to a similar value as $|S_{13}|^2$, which is smaller than $10^{-4}$. In general, all overlaps remain smaller than this value for the $\lambda_{IJ}$ tested. We find that the resulting VMC energy differences, $\Delta E_{12}$, $\Delta E_{13}$, and $\Delta E_{23}$, are similar over a wide range of $\lambda_{IJ}$ (panel b).

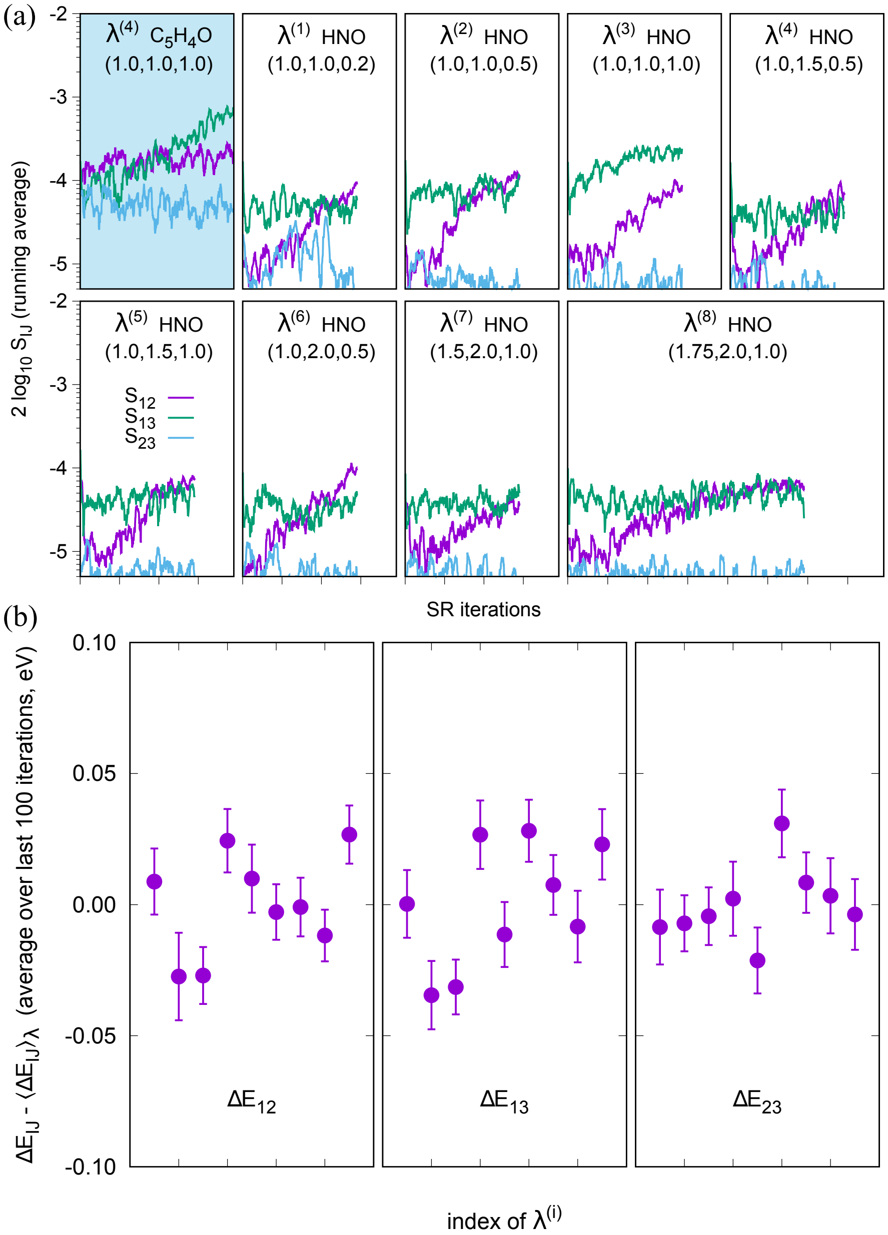


Figure 8: Impact of the choice of $\lambda^{(i)} = (\lambda_{12}, \lambda_{13}, \lambda_{23})$ in a 3-state optimization of nitroxyl on (a) overlaps, $|S_{IJ}|^2$, and (b) excitation energies, $\Delta E_{IJ}$, with respect to the average value, $\langle \Delta E_{IJ} \rangle_\lambda$. The overlaps from a 3-state cyclopentadienone optimization are provided in the first panel of (a) for comparison (see Fig. 2 in the main text). All nitroxyl optimizations are carried out with $\tau_{\rm SR} = 0.025\ {\rm H}^{-1}$ for 600 SR steps. An additional 600 steps in the $\lambda^{(8)}$ calculation are shown to illustrate the eventual flattening of $|S_{12}|^2$. For $\lambda^{(9)} = (2.0, 2.0, 1.0)$, we only show the energy differences but not the overlaps.

## 5.4 Cyclopentadienone overlaps for different determinant expansions

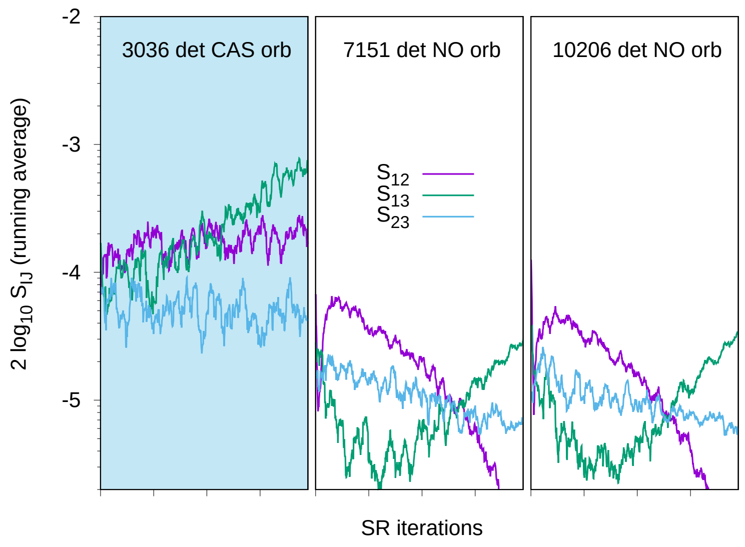


Figure 9: Cyclopentadienone overlaps, $|S_{IJ}|^2$, for three different determinant expansions. All optimizations shown use $\lambda_{IJ} = 1.0$.

In Section 2 of the main text, we discuss in detail the behavior of the overlaps and excitation energies of cyclopentadienone for a small 3036 expansion and different choices of $\lambda_{IJ}$. In Figure 9, we plot the overlaps obtained during the VMC optimization in larger determinant expansions of cyclopentadienone, using $\lambda = (1.0, 1.0, 1.0)$. While this choice of $\lambda_{IJ}$ is not sufficient to keep the overlaps under control for the 3036 expansion, the overlaps for the 7151 and 10206 expansions remain at smaller values throughout the optimization.

# References


(1) Barca, G. M. J.; Bertoni, C.; Carrington, L.; Datta, D.; De Silva, N.; Deustua, J. E.; Fedorov, D. G.; Gour, J. R.; Gunina, A. O.; Guidez, E.; Harville, T.; Irle, S.; Ivanic, J.; Kowalski, K.; Leang, S. S.; Li, H.; Li, W.; Lutz, J. J.; Magoulas, I.; Mato, J.; Mironov, V.; Nakata, H.; Pham, B. Q.; Piecuch, P.; Poole, D.; Pruitt, S. R.; Rendell, A. P.; Roskop, L. B.; Rueden-

berg, K.; Sattasathuchana, T.; Schmidt, M. W.; Shen, J.; Slipchenko, L.; Sosonkina, M.; Sundriyal, V.; Tiwari, A.; Galvez Vallejo, J. L.; Westheimer, B.; Wloch, M.; Xu, P.; Zahariev, F.; Gordon, M. S. Recent developments in the general atomic and molecular electronic structure system. *J. Chem. Phys.* **2020**, *152*, 154102.

(2) Véril, M.; Scemama, A.; Caffarel, M.; Lipparini, F.; Boggio-Pasqua, M.; Jacquemin, D.; Loos, P.-F. QUESTDB: A database of highly accurate excitation energies for the electronic structure community. *Wiley Interdiscip. Rev.: Comput. Mol. Sci.* **2021**, *11*, e1517.

(3) Loos, P.-F.; Boggio-Pasqua, M.; Scemama, A.; Caffarel, M.; Jacquemin, D. Reference Energies for Double Excitations. *J. Chem. Theory Comput.* **2019**, *15*, 1939–1956.

(4) Garniron, Y.; Applencourt, T.; Gasperich, K.; Benali, A.; Ferté, A.; Paquier, J.; Pradines, B.; Assaraf, R.; Reinhardt, P.; Toulouse, J.; Barbaresco, P.; Renon, N.; David, G.; Malrieu, J.-P.; Véril, M.; Caffarel, M.; Loos, P.-F.; Giner, E.; Scemama, A. Quantum Package 2.0: An Open-Source Determinant-Driven Suite of Programs. *J. Chem. Theory Comput.* **2019**, *15*, 3591–3609, PMID: 31082265.

(5) Scemama, A.; Caffarel, M.; Benali, A.; Jacquemin, D.; Loos, P.-F. Influence of pseudopotentials on excitation energies from selected configuration interaction and diffusion Monte Carlo. *Res. Chem.* **2019**, *1*, 100002.

(6) Dash, M.; Moroni, S.; Filippi, C.; Scemama, A. Tailoring CIPSI Expansions for QMC Calculations of Electronic Excitations: The Case Study of Thiophene. *J. Chem. Theory Comput.* **2021**, *17*, 3426–3434.

(7) Loos, P.-F.; Matthews, D. A.; Lipparini, F.; Jacquemin, D. How accurate are EOM-CC4 vertical excitation energies? *J. Chem. Phys.* **2021**, *154*, 221103.

(8) Loos, P.-F.; Lipparini, F.; Matthews, D. A.; Blondel, A.; Jacquemin, D. A Mountaineering Strategy to Excited States: Revising Reference Values with EOM-CC4. *J. Chem. Theory Comput.* **2022**, *18*, 4418–4427, PMID: 35737466.

(9) Saha, B.; Ehara, M.; Nakatsuji, H. Singly and doubly excited states of butadiene, acrolein, and glyoxal: Geometries and electronic spectra. *J. Chem. Phys.* **2006**, *125*, 014316.
